# A Conserved Developmental Patterning Network Produces Quantitatively Different Output in Multiple Species of Drosophila

**DOI:** 10.1371/journal.pgen.1002346

**Published:** 2011-10-27

**Authors:** Charless C. Fowlkes, Kelly B. Eckenrode, Meghan D. Bragdon, Miriah Meyer, Zeba Wunderlich, Lisa Simirenko, Cris L. Luengo Hendriks, Soile V. E. Keränen, Clara Henriquez, David W. Knowles, Mark D. Biggin, Michael B. Eisen, Angela H. DePace

**Affiliations:** 1 Department of Computer Science, University of California Irvine, Irvine, California, United States of America; 2 Department of Systems Biology, Harvard Medical School, Boston, Massachusetts, United States of America; 3 School of Engineering and Applied Sciences, Harvard University, Cambridge, Massachusetts, United States of America; 4 California Institute for Quantitative Biosciences, University of California Berkeley, Berkeley, California, United States of America; 5 Centre for Image Analysis, Swedish University of Agricultural Sciences, Uppsala, Sweden; 6 Genomics and Life Sciences Division, Lawrence Berkeley National Laboratory, Berkeley, California, United States of America; 7 Howard Hughes Medical Institute, University of California Berkeley, Berkeley, California, United States of America; University of California Davis, United States of America

## Abstract

Differences in the level, timing, or location of gene expression can contribute to alternative phenotypes at the molecular and organismal level. Understanding the origins of expression differences is complicated by the fact that organismal morphology and gene regulatory networks could potentially vary even between closely related species. To assess the scope of such changes, we used high-resolution imaging methods to measure mRNA expression in blastoderm embryos of *Drosophila yakuba* and *Drosophila pseudoobscura* and assembled these data into cellular resolution atlases, where expression levels for 13 genes in the segmentation network are averaged into species-specific, cellular resolution morphological frameworks. We demonstrate that the blastoderm embryos of these species differ in their morphology in terms of size, shape, and number of nuclei. We present an approach to compare cellular gene expression patterns between species, while accounting for varying embryo morphology, and apply it to our data and an equivalent dataset for *Drosophila melanogaster*. Our analysis reveals that all individual genes differ quantitatively in their spatio-temporal expression patterns between these species, primarily in terms of their relative position and dynamics. Despite many small quantitative differences, cellular gene expression profiles for the whole set of genes examined are largely similar. This suggests that cell types at this stage of development are conserved, though they can differ in their relative position by up to 3–4 cell widths and in their relative proportion between species by as much as 5-fold. Quantitative differences in the dynamics and relative level of a subset of genes between corresponding cell types may reflect altered regulatory functions between species. Our results emphasize that transcriptional networks can diverge over short evolutionary timescales and that even small changes can lead to distinct output in terms of the placement and number of equivalent cells.

## Author Summary

For a gene to function properly, it must be active in the right place, at the right time, and in the right amount. Changes in any of these features can lead to observable differences between individuals and species and in some cases can lead to disease. We do not currently understand how the position, timing, and amount of gene expression is encoded in DNA sequence. One approach to this problem is to compare how gene expression differs between species and to try to relate changes in DNA sequence to changes in gene expression. Here, we take the first step by comparing gene expression patterns at high spatial and temporal resolution between embryos of three species of fruit flies. We develop methods for comparing gene expression in individual cells, which allow us to control for variation in the size, shape, and number of nuclei between embryos. We find measurable quantitative differences in the patterns for all individual genes that we have examined. However, by considering all genes in our dataset at once, we show that many genes are changing together, leading to largely equivalent types of cells in these three species.

## Introduction

Transcriptional programs specify and elaborate cell identity during animal development, as a single cell gives rise to the hundreds of cell types that comprise the adult animal. Accordingly, variation in the timing, spatial location, and level of transcription is thought to be a major source of molecular variation for morphological changes during evolution [1]–[3]. Gene expression during animal development is highly dynamic in space and time and occurs in the context of a gene regulatory network; the expression of any given gene is dependent on the spatiotemporal expression patterns of many others. This poses a fundamental problem for comparing gene expression patterns between species. Any measured expression differences for a given gene could be due to multiple non-mutually exclusive factors including changes in embryo geometry, changes in the activity, timing or location of expression for upstream regulators or altered regulatory logic, such as no longer responding to a particular regulator. Attributing gene expression differences to these different sources is a fundamental hurdle to employing a comparative approach; overcoming it would allow new types of systematic analyses to address how changes to gene regulation can contribute to new organismal phenotypes.

What strategy can we use to disentangle the potential sources of expression differences? One possibility is to look specifically for regulatory differences in a way that controls for differences in embryo morphology. In the developing embryo, each nucleus must make a decision about whether to express a gene, and to what level. This decision is based on integrating local information about the concentration of upstream regulators, usually DNA binding proteins termed transcription factors (TFs). This regulatory function, termed the input function [4] or gene regulatory function (GRF) [5], relates the concentration of regulators (inputs) to the concentration of their targets (the outputs). If an input function is conserved, we expect to find cells in multiple species with similar concentrations of inputs and outputs, even if they occur in different positions in the respective embryos. We can therefore attempt to identify conserved input functions by identifying cells with similar multi-gene expression profiles. This strategy also provides an embryo-scale view on the output of the gene regulatory network, namely sets of cells distinguished by their transcriptional profile and therefore primed to differentiate into different cell types. Analyzing the set of expression profiles for all cells in the embryo thus reveals how the patterning system allocates cells to different cell types during development.

Comparing cellular gene expression profiles between species requires high-resolution data: specifically, expression measurements for an entire gene regulatory network at cellular resolution in multiple species. Imaging technology now makes it possible to collect quantitative spatiotemporal expression data at cellular resolution for several genes at once. Previously, we developed high-resolution microscopy and image analysis methods to measure gene expression quantitatively in blastoderm embryos of *Drosophila melanogaster (D. melanogaster)*, in roughly 10 minute time intervals during the hour prior to gastrulation [6], [7]. These data are integrated into a gene expression atlas that presents the average expression of many genes in a unified cellular resolution morphological framework. In contrast, most previous comparative gene expression studies in Drosophila have either sacrificed spatial information to obtain quantitative data on many genes using genomic technologies such as arrays or RNA-seq, or used imaging to obtain qualitative spatially resolved data on few genes (for examples in early development of *Drosophila* see [8]–[14]).

**Figure 1:**
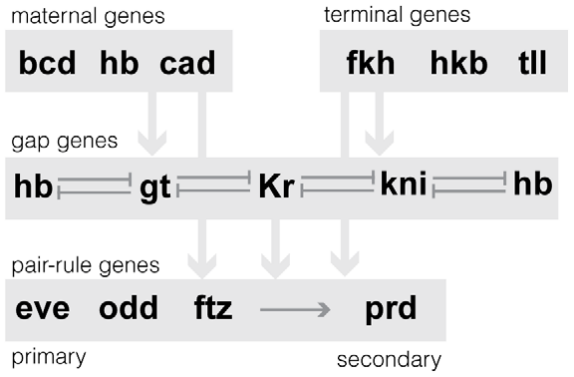
Schematic of the regulatory relationships between 13 AP patterning network genes. In this paper, we examine the expression patterns of a subset of anterior/posterior (AP) patterning genes; general information on the regulatory relationships between the genes in our dataset is shown. Not all regulatory relationships have been precisely defined; therefore, the network is shown as a hierarchy between classes of genes (maternal, gap, terminal and pair-rule) with known interactions within classes. For example, the gap genes are known to cross-repress one another [58], and the primary pair-rule genes are thought to regulate the secondary pair-rule genes [24], [59].

Here, we apply our high-resolution imaging methods to measure gene expression patterns for 13 genes from the segmentation network in blastoderm embryos of two closely related species of *Drosophila*, *Drosophila yakuba (D. yakuba)* and *Drosophila pseudoobscura (D. pseudoobscura)*, and compare our data to a similar pre-existing dataset for *D. melanogaster*. The segmentation network (Figure 1) comprises a small number of well-characterized TFs that interact to generate increasingly complex patterns of gene expression during a short window of early development. The output of the network prefigures the position of the larval segments and associated morphological structures [15]. The TFs and topology of the segmentation network are assumed to be conserved throughout Drosophila, but vary in higher Diptera and other insects including wasps, beetles, mosquitos and bees [13], [16]–[23]. We therefore anticipated that the network output in three closely related Drosophila species would be at least qualitatively similar, but we could not predict *a priori* what type of quantitative differences we would find. We report our findings on quantitative differences in embryo morphology and expression patterns between *D. melanogaster*, *D. yakuba* and *D. pseudoobscura*, our method for comparing cellular gene expression profiles while accounting for changing embryo morphology, and our comparative analysis of cell types at the blastoderm stage of development.

## Results

### Measuring gene expression in Drosophila blastoderm embryos

We used high-resolution microscopy and RNA *in situ* hybridization to image the expression of 13 genes in 616 embryos of *D. yakuba* and 933 embryos of *D. pseudoobscura* (Table S1). This set of 13 genes consists of major determinants of anterior/posterior patterning including the maternal genes bicoid (bcd) and caudal (cad); the gap genes giant (gt), Krüppel (Kr), knirps (kni) and hunchback (hb); the terminal genes forkead (fkh), huckebein (hkb) and tailless (tll); three primary pair-rule genes, even-skipped (eve), fushi-tarazu (ftz), and odd-skipped (odd); and one secondary pair-rule gene, paired (prd) [24]. Staining for cad in *D. pseudoobscura* was consistently low-level and uniform, and is therefore not included in the dataset. While antibodies are available for some of the components of the network, they vary widely in quality and work with different efficiencies in different species. Where protein levels have been measured in *D. melanogaster*, they correlate well with RNA levels except in notable cases, such as hunchback, where translational control is known to play a role [6].

Each embryo was stained for the gene of interest, a DNA dye and a second gene serving as a fiduciary marker. Embryos were manually staged into 6 time intervals spanning the hour prior to gastrulation by assessing the extent of cell membrane invagination under phase contrast illumination. Embryos were then imaged using 2-photon microscopy, and the resulting image stacks were segmented to generate individual pointcloud files, which record the 3D location and gene expression values associated with each nucleus [6]. Pointcloud files for individual embryos were registered together to produce gene expression atlases for *D. yakuba* and *D. pseudoobscura*. In these atlases, average expression for all of the genes in our dataset are present in a species-specific dynamic morphological framework based on cellular density patterns. Expression levels within an atlas are normalized per gene with expression levels scaled so that the time point with the highest expression value takes on a value of 1. For a detailed description of atlas building methods, see [7].

**Figure 2:**
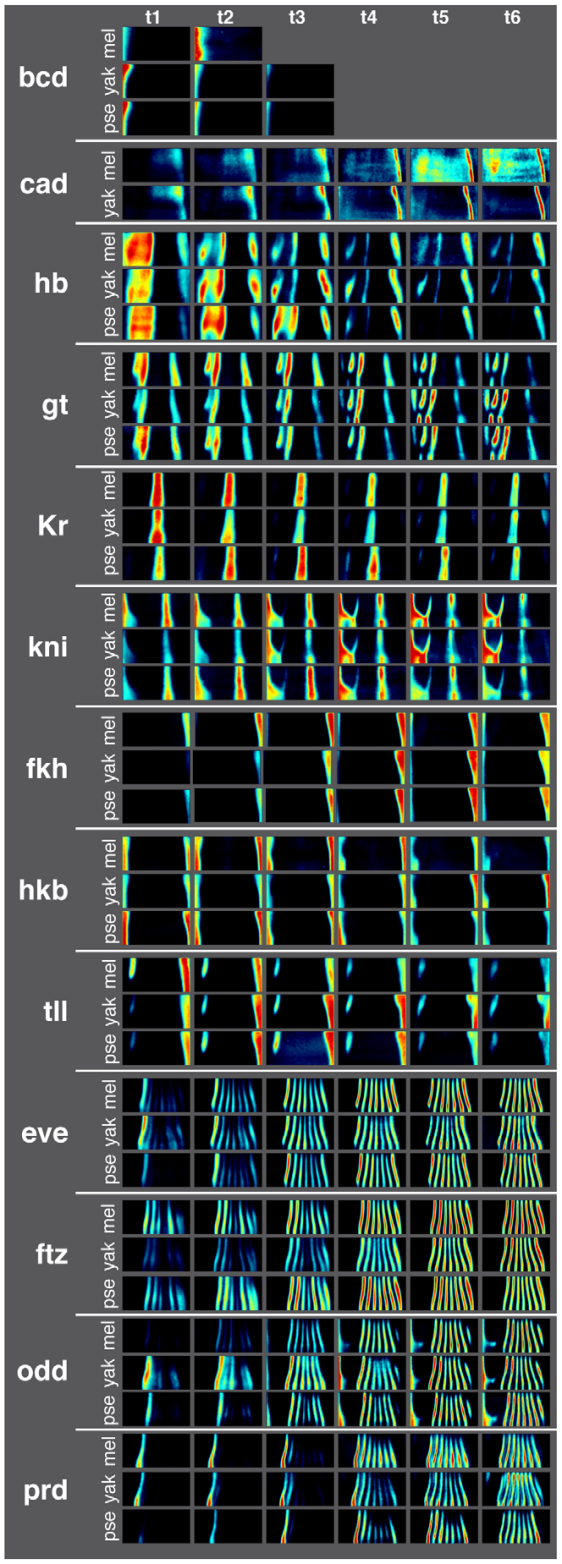
Average gene expression patterns for 13 AP patterning genes in *D. melanogaster*, *D. yakuba*, and *D. pseudoobscura* are qualitatively similar. Fluorescent *in situ* hybridization, 2-photon microscopy and image analysis were used to measure the expression of 13 AP patterning genes at cellular resolution in *D. melanogaster* (see [7]), *D. yakuba* and *D. pseudoobscura* over 6 time points during the hour prior to gastrulation. Because the embryo is bilaterally symmetric, one half of a cylindrical projection (an “unrolled” embryo, dorsal side up and anterior to the left) is shown for each time point. High expression is red; low expression is black. Bcd is not expressed during the last three time points in *D. yakuba* and *D. pseudoobscura* and therefore not shown. Staining for cad was consistently low level and uniform in *D. pseudoobscura* and therefore not included in the dataset.

Average patterns for each gene for the six time intervals in our dataset are shown in Figure 2 alongside the corresponding genes in the reference *D. melanogaster* dataset [7]. We assessed the quality of the data by two measures, the range of intensities measured for a given gene, which reflects the ratio of signal to noise (Figure S1), and the average standard deviation in expression after registration (Table S2). The atlases for *D. yakuba* and *D. pseudoobscura* are of similar quality to the previously assembled *D. melanogaster* dataset.

### Species differ in blastoderm embryo morphology

**Figure 3:**
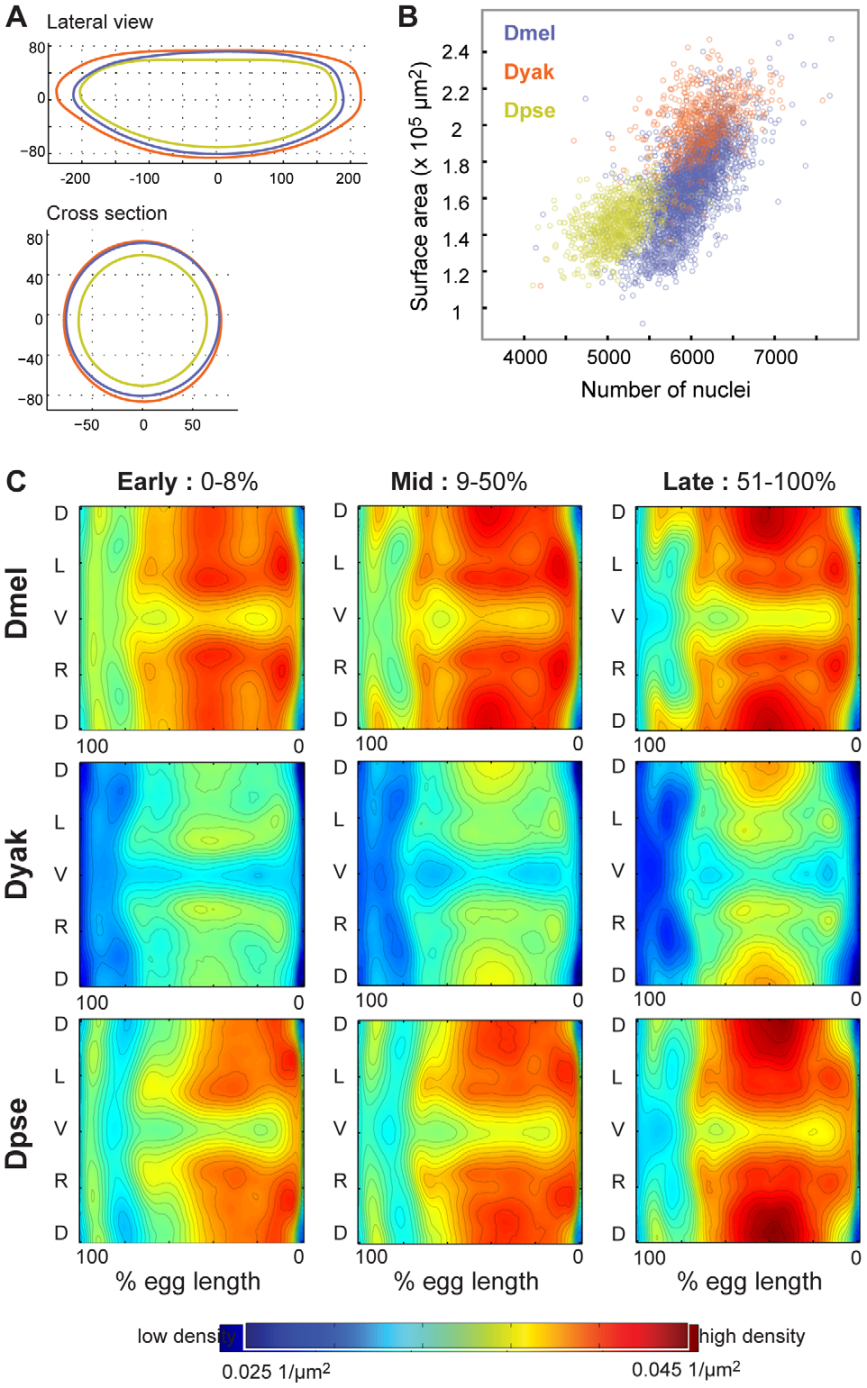
Blastoderm embryos of the three Drosophila species vary in size, shape, and number of nuclei. (A) Silhouettes of species-specific embryo models are shown in both lateral view (anterior left, dorsal up,) and cross section (dorsal up), *D. melanogaster* (blue), *D. yakuba* (orange), *D. pseudoobscura* (green). (B) For each embryo in our datasets, the surface area was calculated and compared to the number of nuclei. Though both of these values vary within and between species, the relationship between them is linear. (C) Density patterns for early, middle and late stage blastoderm embryos are displayed as heat maps (red is high density, blue is low density), with corresponding contours on 2D cylindrical projections of the embryos. Anterior is to the left, posterior to the right. D = dorsal, L = left, V = ventral, R = right. In pairwise comparisons (Figure S2) the densities are statistically distinct across all three species with *D. melanogaster* and *D. pseudoobscura* containing the largest areas of similar nuclear density.

Though qualitatively similar, our data revealed several quantitative morphological differences between *D. melanogaster*, *D. yakuba* and *D. pseudoobscura* embryos including differences in blastoderm shape, size and the number of nuclei (Figure 3). These differences required us to build species-specific atlases to account for the different embryo morphologies, rather than register all data into a single morphological framework. Comparison of the eggs of the three species revealed that they vary both in their anterior/posterior shapes (compare *D. yakuba* to *D. melanogaster* and *D. pseudoobscura*, Figure 3a), and their circumferences (compare *D. yakuba* and *D. melanogaster* to *D. pseudoobscura*, Figure 3a). Ordering the embryos in terms of average egg length or surface area, *D. pseudoobscura* embryos are the smallest, followed by embryos of *D. melanogaster* and *D. yakuba* (Figure 3b, Table 1). Notably, the number of nuclei scales linearly with surface area within each species with the same relationship (slope) (Figure 3b). However, this doesn't completely explain changes in nuclear number between species, as even some embryos with the same surface area have different numbers of nuclei (note in particular differences between *D. pseudoobscura* and *D. melanogaster* embryos).

**Table 1:** Egg length and nuclear number varies in blastoderm embryos of *D. melanogaster*, *D. yakuba*, and *D. pseudoobscura*.

Species	Embryos	Ave. No. Nuclei	Std Dev	Ave. egg length (µm)	Std Dev
*D. melanogaster*	2772	5974.1	339.12	393.8	30.75
*D. yakuba*	618	6127.8	348.13	451.8	22.74
*D. pseudoobscura*	932	5086.7	327.39	394.6	18.84

The number of embryos in each atlas is shown, along with the average number of nuclei and average egg length, with associated standard deviations (*D. melanogaster* data from [7]).

Nuclear density patterns prefigure movements during gastrulation [25], [26]. We found that the spatial patterns of local nuclear density are similar between the three species (Figure S2), though the average density of nuclei on the surface of *D. yakuba* embryos is lower than that for the other two species (Figure 3c). The overall similarities between the species' nuclear density patterns, including lower density around the cephalic furrow and along the ventral midline, indicate that nuclear density patterns likely reflect conserved developmental processes.

During cellularization, nuclei move from the poles towards the center and this can contribute to shifts in gene expression patterns. We call this “cell flow” to distinguish it from “expression flow” [26]. The overall direction and magnitude of cell flow movements are similar between these 3 species (Figure S3). A species-specific model of cell flow based on changing density patterns is used to find corresponding cells across time points during atlas construction. As a result, comparison of cellular gene expression profiles over time between the expression atlases removes the effect of differences in cell flow [7], [27].

### Expression distance can be used to compare cellular gene expression profiles

To systematically analyze expression differences in this transcriptional network, we developed a method to compare gene expression profiles on a cell-by-cell basis. Each cell's gene expression profile can be represented as a vector whose entries are defined by the average expression level for a given gene at a given time point. We used the squared Euclidean distance between such vectors to score the difference between any two cells; we call this the *expression distance score*. We used the squared distance (rather than the Euclidian distance) because it is additive across genes and time points which makes interpretation of the contributions of each gene to the overall expression distance simple to interpret. The expression distance score can be calculated based on any subset of genes in the dataset including single genes, groups of specific interacting genes, or the entire dataset simultaneously. These analyses are possible because our dataset contains expression levels measured for multiple genes in the same cellular resolution framework.

**Figure 4:**
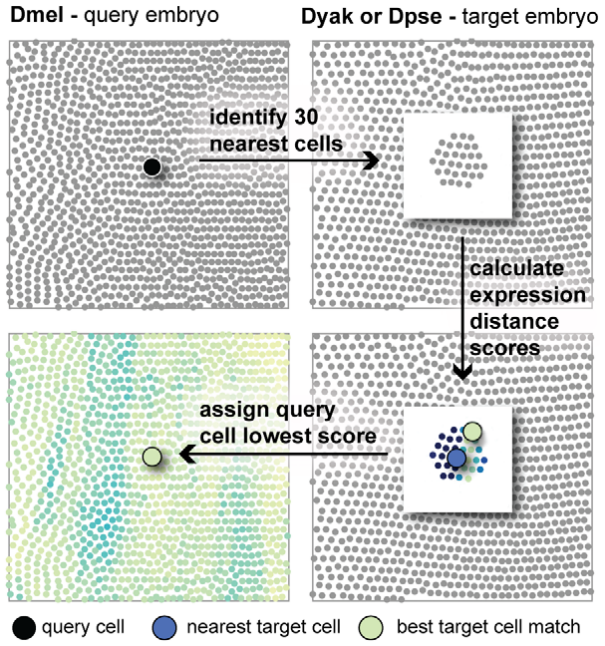
The expression distance metric can be used to search for corresponding cells. A schematic of the algorithm to identify corresponding cells is shown. For a given query cell, the 30 nearest cells in 3D space in the target embryo are identified. The expression distance between the query cell and each of these target cells is calculated. The best corresponding cell is the target with the lowest expression distance score. This is often not the target cell nearest to the query cell in 3D space.

Comparing gene expression in this way has several advantages over standard methods where gene expression patterns are compared individually in terms of morphological features of the embryo such as relative egg length. First, this method doesn't rely on choosing an arbitrary threshold for deciding whether a cell is “expressing” or not. Choosing thresholds is particularly problematic for genes with graded expression patterns such as the gap genes. Second, the expression distance score makes use of the whole expression level time course while factoring out the effects of morphological movements (i.e. cell flow). Additionally, the expression distance score can be used as a natural criterion for selecting cells amongst a set. For example, to find cells with similar expression profiles near to a given query cell, one could first define a set of nearby cells to search, then calculate the expression distance score for the query cell compared to each cell in the set. The best match will have the lowest expression distance score (Figure 4). We use the expression distance score to compare the expression profiles of cells that are spatially nearby both within and between species to determine how expression patterns differ in terms of their output, relative location in the embryo, and the relative number of expressing cells.

Assessing statistical significance of the expression distance score directly is difficult since it is based on multivariate quantities whose correlations we have not measured; this would require co-staining every pair of genes in our dataset. We provide two methods to gauge significance. First, we constructed two atlases of *D. mel* expression from disjoint sets of embryos. Each of these atlases was assembled from approximately the same number of individual embryos per gene as the *D. yakuba* and *D. pseudoobscura* atlases. Expression distances between cells in these two atlases provide a baseline for what should be insignificant expression distance scores with respect to measurement error and intra-strain variability. Second, we analyzed differences in expression level for each gene and time point independently using a two-sample t-test (see Materials and Methods). For a pair of cells in two different atlases, we can determine whether a gene's expression is significantly different relative to the variance across measurements of that gene and time point. We can then declare a pair of corresponding cells to be different if they have significantly different expression levels of one or more genes at one or more time points, applying a suitable correction for multiple hypothesis testing. We tallied the number of entries in the expression profile that are statistically different, and call this the t-test score. It is more conservative than the expression distance since it doesn't detect the sum of many small differences across multiple genes or time points. However, it does provide a simple model of statistical significance, validating that the average expression differences we observed are significant relative to error in our measurements.

### Expression of individual genes differs in dynamics and relative position

Subtle differences in the dynamics of expression patterns are detectable from inspecting the averaged, normalized expression patterns of all genes in the dataset (Figure 2). The peak of expression varies between species for multiple genes (note Kr, fkh, hkb and ftz). For some patterns with multiple domains, such as eve, the relative level of the different stripes varies between species. Finally, some patterns also vary differently over the dorsal/ventral axis (note the longest anterior stripe of gt in later time points).

**Figure 5:**
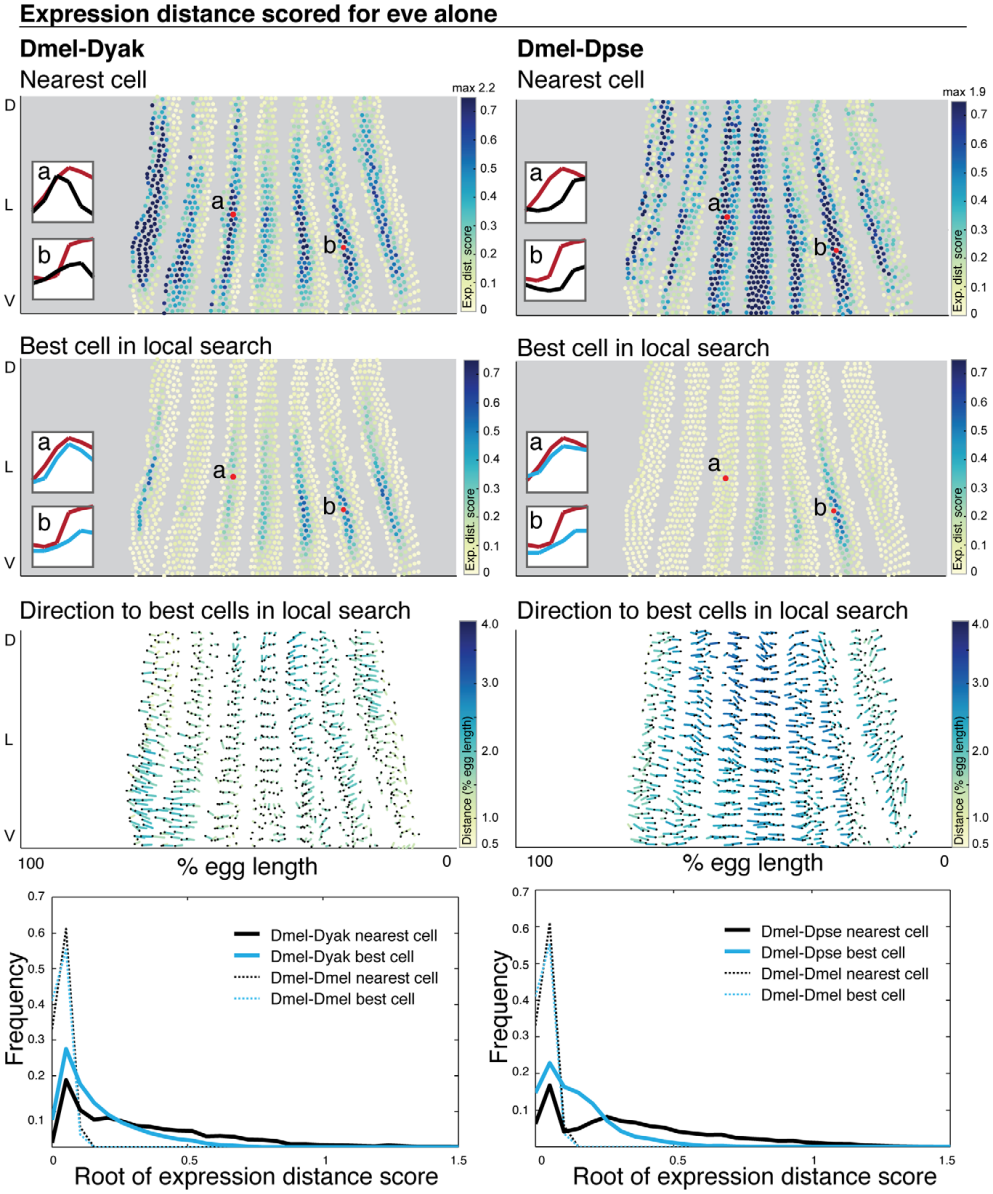
Even-skipped expression varies in relative position and intensity. The expression distance score for each cell is plotted on a 2D representation of the embryo and the underlying gene expression profiles are illustrated in insets where gene expression is represented as a line trace over time. (1st row) For each *D. melanogaster* query cell, the expression distance score of the nearest target cell in *D. yakuba* (left) and *D. pseudoobscura* (right) is shown. (2nd row) The expression distance score for the best matched cell within the nearest 30 cells for both *D. yakuba* and *D. pseudoobscura* is shown. High expression distance scores, indicating poor matches, are darker. All cells scoring above 0.7 are colored the darkest blue; when the maximum value exceeds 0.7, the maximum value amongst all cells is reported at the top of the color map. Representative *D. melanogaster* cells are labeled, and their expression profiles are shown in the insets compared to their matches in *D. yakuba* or *D. pseudoobscura* (*D. melanogaster* cell in red, *D. yakuba* or *D. pseudoobscura* cell in blues - dark blue for nearest cell, light blue for best cell after local search). For each representative *D. melanogaster* cell, we list the label in the figure (a or b), the cell ID number, the target embryo to which it was matched (*D. yakuba* or *D. pseudoobscura*) and the expression distance score to the nearest target cell and the best matched target cell: a. 4314, *D. yakuba*, 0.719, 0.141; b. 5232, *D. yakuba*, 0.633, 0.557; a. 4314, *D. pseudoobscura*, 0.611, 0.066; b. 5232, *D. pseudoobscura*, 0.966, 0.524. (3rd row) For each *D. melanogaster* query cell, the distance and direction to the average position of the top 10 best corresponding target cells is shown. The correspondence is shown with a line that starts at the position of the query cell, and ends at the average position of the target cells. The end of the line is indicated with a black dot. Because the 2D projection distorts actual distance in 3D, the lines are color-coded to indicate actual distance traversed in 3D. Dark blue is a large distance, yellow is a small distance. (4th row) The distribution of expression distance scores using only the nearest cell (grey) and best-matched cell within the nearest 30 (blue) are shown; we plot the root of the expression distance score to separate values near zero. The distribution of expression distance scores narrows and the mean and median decrease after a local search (Table S3). To establish the significance of the calculated differences, we assembled two atlases from the *D. melanogaster* dataset, and compared these two atlases to each other (dotted lines).

To systematically analyze variation in each gene's expression pattern, we calculated the expression distance score for each cell in *D. melanogaster* compared to its spatially nearest cell in *D. yakuba* or *D. pseudoobscura*, for each gene in our dataset, one at a time. Because embryos are of different sizes, we scaled each embryo to the same relative egg length and aligned atlases by their centers of mass before determining spatial relationships between cells. To determine if there are positional shifts in expression patterns, we then performed a local search amongst the 30 spatially nearest cells for the cell with the best match to the *D. melanogaster* expression profile. This corresponds to movement by 3–4 cells in any direction. We did do not require a one-to-one match; instead we allowed multiple query cells to match the same cell in the target species. This flexibility was necessary because of the differing numbers of cells between the species; forcing a one-to-one match would give misleadingly large expression differences for cells that have clear counterparts in the target embryo, but too few of them. To visualize the results of the search, we assigned the query cell the score of its best match. The breadth of our dataset prevents us from presenting all of these results in the main text of this paper. For this analysis, and the others described below on single gene expression profiles, we show representative data from even-skipped in Figure 5 and the remaining data is presented in Figure S4. This data can also be viewed using our interactive visualization tool, MulteeSum (see Materials and Methods).

A local search improved the expression distance score for most *D. melanogaster* cells as compared to a direct spatial mapping, indicating that eve expression patterns have shifted in space (Figure 5). This also holds for the t-test score (Figure S5). More generally, this is true of all other genes in our dataset, where the mean expression distance score decreases 2 to 5-fold using local search, indicating widespread shifts in relative position (Figure S4, Table S3). To visualize the direction of positional shifts in expression, we determined the distance and direction to the average position of each cell's top 10 hits (Figure 5, Figure S4). Expression of eve is shifted anteriorly for some stripes in *D. yakuba*, while it is shifted posteriorly for all stripes in *D. pseudoobscura*. This is consistent with more conventional representations such as plotting stripe boundaries for specific time points, which also show significant differences in the relative position of eve stripe boundaries (Figure S6). The direction of movement is roughly similar across most genes, with the exception of the terminal genes, where the movement is towards the poles; there is a partial anterior shift for many *D. yakuba* genes and a pronounced posterior shift for nearly all *D. pseudoobscura* genes (Figure S4).

Not all cells have a perfect match in the other species, as indicated by higher expression distance scores even after a local search. For eve, cells in the middle of some stripes differ in their dynamics and relative level (Figure 5, cells labeled b). Differences of this sort are apparent at all tiers of the network (Figure S4). This analysis is an underestimate of expression differences because we do not force one to one matching; there are thus some cells in *D. yakuba* and *D. pseudoobscura* that are not matched. We analyzed the number of matched cells in *D. yakuba* and *D. pseudoobscura* (Figure S7 and MulteeSum, see Materials and Methods), and found that most (>85%) *D. yakuba* and *D. pseudoobscura* cells appeared in the top 10 matches to at least one *D. melanogaster* cell. Furthermore, unmatched cells were distributed spatially almost exclusively in areas where eve is not expressed, indicating that there are not large populations of unmatched cells in *D. yakuba* and *D. pseudoobscura* that are significantly different than their matched neighbors.

### Most cellular gene expression profiles are broadly but not precisely conserved

From the analysis of individual genes, we learned that the relative position of many genes has shifted and that there are some differences in relative levels and dynamics. To assess whether these differences are due to positional shifts in the expression of multiple genes or changes in input functions, we compared gene expression profiles for multiple genes in our dataset simultaneously. Consider the case where the expression pattern of one gene has shifted in space. If this change in expression (the output) is due to a change in the position of an upstream regulator, we would expect the cell's gene expression profile to remain the same. If it is due to a change in the gene's input function (i.e. it is responding to an upstream input differently), we would expect a difference in the concentration of inputs relative to outputs; in other words, a change to the cell's gene expression profile.

For cases where the regulatory relationships between inputs and output are well defined, the relation between expression patterns and the input function can be modeled and tested directly. We have undertaken this type of analysis for expression of the hunchback posterior stripe in a parallel study (Z. Wunderlich et al., submitted). However, the segmentation network is highly interconnected [28] and not all regulatory relationships have been identified. We therefore calculated the expression difference score for all genes in our dataset simultaneously to assess the extent of regulatory differences across the segmentation network in an unbiased, exploratory manner. Cells with differences in cellular gene expression profiles reveal potential regulatory differences. However, these differences are not attributable to any particular input function without further analysis.

**Figure 6:**
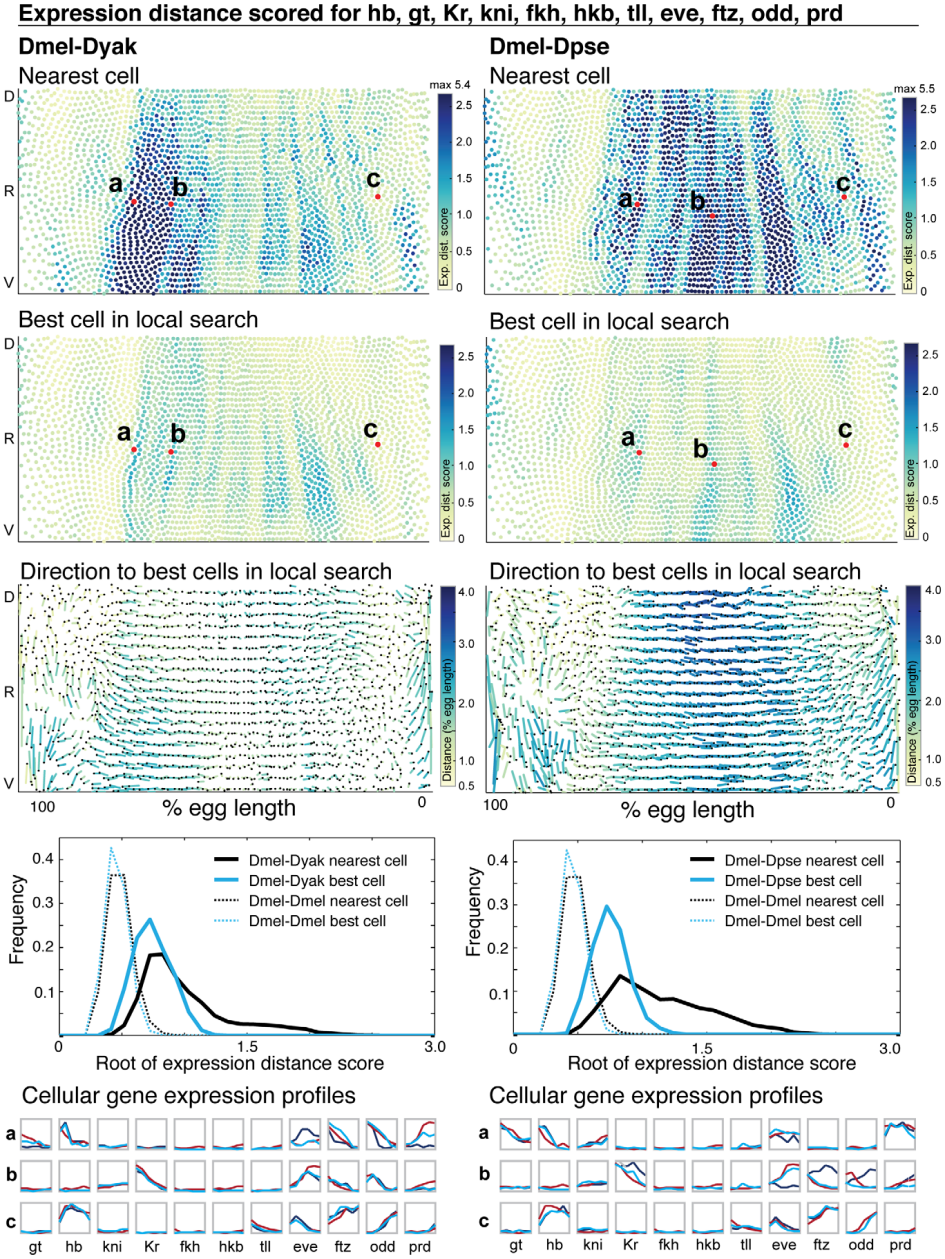
The majority of cellular gene expression profiles are conserved. The expression distance score for each cell is plotted on a 2D representation of the embryo and the underlying gene expression profiles are illustrated in graphs below, where gene expression is represented as a line trace over time for each gene in the dataset. (1st row) For each *D. melanogaster* query cell, the expression distance score of the nearest target cell in *D. yakuba* (left) and *D. pseudoobscura* (right) is shown. (2nd row) The expression distance score for the best matched cell within the nearest 30 for both *D. yakuba* and *D. pseudoobscura* is shown. The same representative cells from the top panel are indicated. High expression distance scores, indicating poor matches, are darker. All cells scoring above 2.5 are colored the darkest blue; when the maximum value exceeds 2.5, the maximum value amongst all cells is reported at the top of the color map. Representative *D. melanogaster* cells are labeled, and their expression profiles are shown in detail at the bottom. For each representative cell, we list the label in the figure (a, b, or c), the target embryo to which it was matched (*D. yakuba* or *D. pseudoobscura*) and the expression distance score to the nearest target cell and the best matched target cell: a, *D. yakuba*, 3935, 3.374, 1.018; b, *D. yakuba*, 4583, 0.881, 0.683; c, *D. yakuba*, 5644, 0.416, 0.355 (the nearest cell appears in the top 10 matches for this cell); a, *D. pseudoobscura*, 3630, 0.884, 0.712; b, *D. pseudoobscura*, 4583, 5.264, 0.811; c, *D. pseudoobscura*, 5644, 0.595, 0.529 (the nearest cell is the best match for this cell). (3rd row) For each *D. melanogaster* query cell, the distance and direction to the average position of the top 10 best corresponding target cells is shown. The correspondence is shown with a line that starts at the position of the query cell, and ends at the average position of the target cells. The end of the line is indicated with a black dot. Because the 2D projection distorts actual distance in 3D, the lines are color-coded to indicate actual distance traversed in 3D. Dark blue is a large distance, yellow is a small distance. (4th row) The distribution of expression distance scores using only the nearest cell (grey) and best-matched cell within the nearest 30 (blue) are shown; we plot the root of the expression distance score to separate values near zero. The distribution of expression distance scores narrows and the mode decreases after a local search (Table S3). To establish the significance of the calculated differences, we assembled two atlases from the *D. melanogaster* dataset, and compared these two atlases to each other (dotted lines). (5th row) The expression profiles of the representative cells labeled in the top and middle panels are represented as a series of chart maps [57], [60] where each gene is a single box with a line trace indicating expression over time. All gene expression data is normalized to a maximum of 1.0 over the time course. The expression profile of the *D. melanogaster* query cell is shown in red, the cell in the target embryo (*D. yakuba* or *D. pseudoobscura*) is shown in blue (dark blue for the nearest cell, and light blue for the best cell after a local search).

Figure 6 shows the expression distance metric calculated using all genes in our dataset except for bcd and cad (see Materials and Methods). As we did for matching cells based on single gene expression profiles, we searched locally amongst the nearest 30 cells for the best match to the query cell, and did not require a one-to-one match. We confirmed that our matching protocol is not missing large numbers of cells in *D. yakuba* and *D. pseudoobscura* (>99.5% matched), and that unmatched cells are intermingled with matched cells (Figure S8 and MulteeSum, see Materials and Methods). We again found that a local search significantly decreases the expression distance score relative to direct spatial mapping for many cells, with the mean decreasing by 2-fold (Figure 6, Table S3). This reflects the shifted relative position of expression for many genes in both species. Visualizing the direction of each pair-wise match reveals areas where corresponding cells are shifted along the anterior/posterior or dorsal/ventral axis; frequently they are shifted along both axes, and the patterns are consistent with those observed for individual genes. These positional shifts are particularly uncoordinated in the ends of the embryos, where corresponding cells are found both closer and further away from the ventral midline. This may be in part because the atlases are assembled by registration using pair-rule genes whose expression is confined to the trunk, and hence our expression data is less accurate at the poles [7]. In the trunk region, there is a pronounced anterior shift of *D. yakuba* cells relative to *D. melanogaster* in the anterior, and a pronounced posterior shift of *D. pseudoobscura* cells relative to *D. melanogaster* throughout the eve expressing region. The genes that are expressed in the trunk are highly interconnected; most regulate one another and would therefore be expected to move together. From the variety of positional shifts observed, we conclude that these expression differences are not likely to result from simple changes in the maternally driven morphogens bicoid or caudal, in which case we would expect coordinated positional shifts along a single axis. Instead, our data is consistent with many small-scale changes throughout the network.

After searching locally, the majority of *D. melanogaster* cells do not differ from their corresponding cells in *D. yakuba* in more than five of their expression profiles' entries, or in *D. pseudoobscura* by more than seven (Figure S9). This indicates that most expression differences we observe for individual genes are attributable to coordinated positional changes in the network as a whole. For some *D. melanogaster* cells, the best match still exhibits some expression differences according to the expression distance metric (Figure 6). The expression distance metric could be high in these cases due to large differences in expression for a single gene, or small differences in many genes. By examining the underlying gene expression profiles for the cells with the highest expression distance metric, we find the latter to be true; the differences that remain after local matching are due to quantitative changes in dynamics and relative levels of expression for multiple genes, rather than the presence or absence of a particular gene product (Figure 6 and MulteeSum, see Materials and Methods). Because the expression distance score is additive, we can assess which genes contribute to the overall score by calculating the expression distance score for each individual gene as well as relevant subsets (Figure S10). We find that differences are widespread; they are not confined to a single gene or tier of the network. The t-test score reveals good concordance between those cells that have large expression distance and those that differ significantly in many individual expression measurements (Figure S9).

In both analyses, *D. yakuba* is more similar to *D. melanogaster* in terms of gene expression profiles. This is plausible as *D. melanogaster* and *D. yakuba* are more closely related than *D. melanogaster* and *D. pseudoobscura* [29]. Some of the small expression differences we identify using the expression distance metric undoubtedly represent experimental noise, but some may represent bona fide regulatory differences between these species. Notably, our expression distance metric identifies cells with differing expression of odd and prd between *D. melanogaster* and *D. yakuba*, and both odd and prd exhibit differential binding of hb in *D. yakuba*, as measured by Chip-Seq [30]. Together with our data, this indicates potentially altered input functions for these genes. Verifying candidate regulatory differences will require assigning them to specific input functions, and functional studies to determine the mechanistic basis of the regulatory change.

### Equivalent cell types occur in different relative proportions between species

At this stage of development, cells are still morphologically similar and yet are committed to their future fates as components of larval structures [15]. Their fate is highly correlated with their spatial position in the embryo and is determined by the set of genes that they express. Therefore, we consider gene expression profile to be equivalent to cell type at this stage. Even if all cell types had precisely equivalent gene expression profiles, they could give rise to morphological differences between embryos if they occur in different relative locations, or in different proportions between embryos. In the previous section, we established that equivalent cell types occur in different relative locations in these three species. Because the embryos also have different numbers of nuclei, a natural question is whether they allocate cell types proportionally.

**Figure 7:**
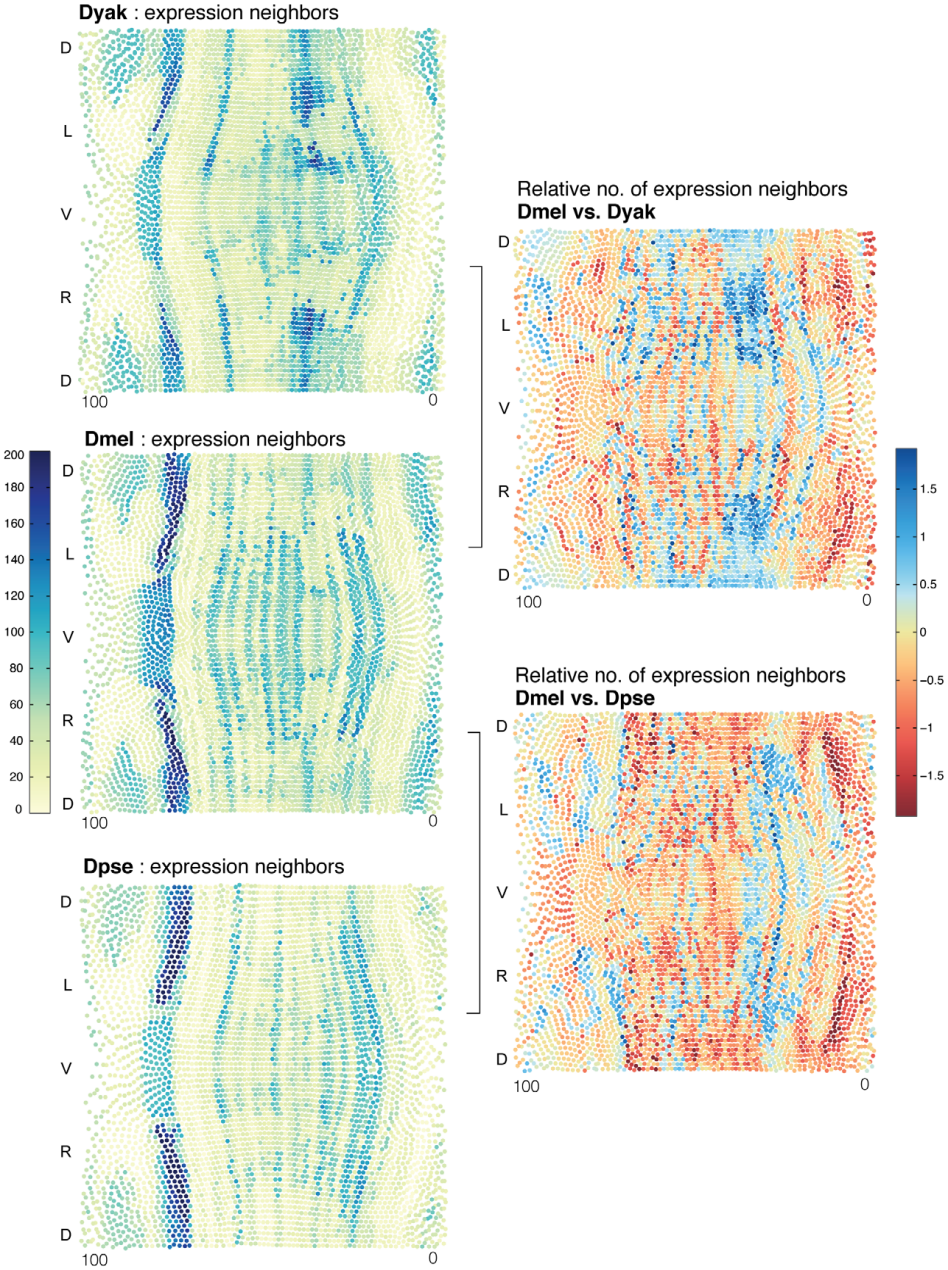
The proportion of cell types varies between *D. melanogaster*, *D. yakuba*, and *D. pseudoobscura*. (Left) To compare the allocation of cell-types across species, we calculated the expression distance score to every other cell in each species' atlas (left). The number of adjacent cells below a given expression distance score is a measure of the size of a neighborhood of similar cells; we call these cells “expression neighbors.” Cells with many expression neighbors are blue. Cells with fewer are yellow. These sets of expression neighbors per cell provide a means to compare the relative allocation of cell types in the different embryos. For each *D. melanogaster* cell, the number of *D. melanogaster* expression neighbors is compared to the number of expression neighbors for its best corresponding cell in *D. yakuba* and *D. pseudoobscura* (right). Values displayed are the log ratio of the neighborhood sizes for corresponding cells in the two species being compared. *D. melanogaster* cells with relatively more expression neighbors are blue. *D. melanogaster* cells with relatively fewer are red.

One possible solution to analyzing cell types would be to cluster the cells based on expression profile and count the number of cells within each cluster. However, expression is changing in a graded way at almost every point in the embryo making it difficult to decide how many clusters there should be. Instead of using an arbitrary clustering of cells, we determined how many adjacent cells are similar to a given query cell by counting how many adjacent cells are within a given expression distance score. This connected set of “expression neighbors” is therefore a group whose expression profile is quantitatively similar to the chosen cell. For any fixed threshold imposed on the expression distance score, the size of this neighborhood captures how quickly expression levels change in the vicinity of a cell. We visualize how this neighborhood size varies over the surface of the embryo (see Figure 7, left). Large neighborhoods correspond to regions of roughly constant cell type. Thus, the number of expression neighbors per cell provides a means to compare allocations of cells between different species on a cell-by-cell basis. If variations in expression between different species reflected a simple uniform scaling, then the neighborhood size for every nucleus would also be proportionally smaller or larger by the same scale factor. On the other hand, if the patterning network of one species allocates a relatively larger population of cells to a given type in some region of the embryo, then the local neighborhood for each of the cells in that region will grow larger.

We calculated the relative expansion or shrinkage of each cell neighborhood between corresponding best-matched cells in *D. melanogaster*, *D. yakuba* and *D. pseudoobscura*. The most important observation from this analysis is that the relative proportion of cells in these expression neighborhoods varies both up and down by as much as 5 fold (Figure 7). These three species allocate cell types quite differently; there are discrete areas of relative expansion and contraction. For example, there are relatively more *D. melanogaster* cells than their equivalents in *D. yakuba* and *D. pseudoobscura* in the posterior trunk, (roughly corresponding to the position of the last 3 stripes of even-skipped expression), but fewer immediately posterior, on the border of terminal gene expression. We conclude that small changes to the behavior of the patterning network, achieved either through quantitative regulatory changes, or by initiating patterning in a new morphological context, or both, can result in different proportions of cells allocated to conserved cell types. These could serve as the initial basis for downstream morphological changes.

## Discussion

Here, we have compared the mRNA expression of 13 developmental regulatory genes in blastoderm embryos of *D. melanogaster*, *D. yakuba*, and *D. pseudoobscura* using species-specific atlases where average relative expression levels for all genes in the data set are present in the same cellular resolution morphological framework. These atlases provide a comprehensive quantitative view of a developmental gene regulatory network operating in closely related species and show that all genes examined show clear quantitative differences in expression pattern between these species. By searching locally for cells with similar gene expression profiles, we filtered out expression differences due to changing morphology and those due to positional changes in upstream regulators. This analysis demonstrated that cells of similar types are conserved between these species, though they differ in terms of their relative position and proportion. The analysis also revealed a minority of cells potentially using different input functions.

### Applications for comparison of cellular resolution data

Identifying the genetic differences that cause variation in gene expression is a major goal not only for evolutionary developmental biologists, but also for those interested in human disease. An increasing number of disease associated variants have been mapped to regulatory regions of the genome [31]; to contextualize their effects we must learn which sequence variants are likely to alter gene expression and which will not. The approach we describe identifies candidate regulatory differences from cellular resolution data on a network of interacting genes. To obtain data for all of the relevant genes over time, we built averaged atlases of gene-expression using high-resolution imaging and registration techniques. This type of data is likely to become increasingly common as these technologies continue to improve. For example, live *in-toto* imaging techniques such as SPIM have been successfully applied to blastoderm embryos and are likely to provide a view of the behavior of the network at much higher temporal resolution [32], [33]. As sequencing methods become more sensitive, they may also be able to generate spatially resolved data by either separating cells for biochemical analysis or using imaging-based methods to sequence transcripts *in situ* [34]. We therefore anticipate that increasing numbers of studies will involve comparing spatially resolved cellular resolution gene expression profiles between different samples from different species, different populations, or from the same individual under different conditions. The expression distance metric is a useful tool to focus attention on subsets of interacting components that are likely to show different behavior between species. Such methods may be applied to less well-characterized gene regulatory networks, where unbiased methods for reconstructing gene regulatory networks and mapping expression differences onto a network from a combination of genomic and functional data will be required.

### Comparing multi-gene expression profiles reveals candidates for regulatory change

A grand challenge in the post-genomic era is how to move from broadly identified expression differences to precise identification of mechanistic differences in the underlying gene regulatory networks. Regulatory divergence across multiple scales, from the topology of the network to fine-scale changes in input functions, has been observed in comparative studies of the Ascoycota fungi [35] and animals [36]. In principle, the quantitative differences in gene expression we observe could result from many non-mutually exclusive components of the gene regulatory network, including changes in trans-acting TFs (either in their DNA binding affinity or in their interaction with other components), *cis*-regulatory modules (CRMs), chromatin structure, promoter architecture, or transcript stability. Even at the short evolutionary distances studied here, genetic changes are observed in multiple tiers of this developmental network. DNA binding domains of TFs are highly conserved, with only single amino acid changes in some lineages, but the remainder of the protein diverges more rapidly [29]. *cis*-regulatory sequences that interpret the concentrations of these TFs differ substantially in terms of the number, affinity and arrangement of TF binding sites [37]. Notably, a recent comparative study of the dorsal/ventral patterning network in blastoderm embryos of Drosophila showed that changes in the arrangement of TF binding sites in CRMs leads to quantitative gene expression differences between species by altering input functions [38]. Other relevant features such as chromatin structure, promoter architecture and miRNAs have recently been systematically functionally characterized in *D. melanogaster* [39]–[41], laying the foundation for future comparative studies.

Attributing expression differences to these features will require a model of the system to generate experimentally verifiable hypotheses. There are an increasing number of models that take advantage of spatially resolved expression data and knowledge of TF binding sites to predict CRM output [42]–[45]. However, these models do not predict expression accurately enough to capture the quantitative differences we observe between species. Our high-resolution expression data are well suited to the development of new types of models for ascertaining the source of expression differences, a clear line of future experimentation.

### Expression output in the segmentation network is robust to genetic and morphological change

Despite the quantitative differences in cellular gene expression patterns that we measure, the segmentation network produces remarkably similar cell type output in the face of substantial genetic and morphological perturbation. This implies that formation of these cell types is under strong selective constraint. *D. melanogaster* embryos can tolerate variation in the proportion of cell types, though there is an upper limit on how much the patterning system can be compressed [46]. The differences we observe may reflect neutral drift within these limits. For example, there may be restrictions on nearly neutral processes of binding site turnover, where small sequence changes cause quantitative variation in output, and subsequently require fine-tuning of expression to stay within acceptable limits [47]. This sort of process would result in fine-scale expression changes as the acceptable limits are explored. Because many expression patterns are qualitatively conserved between closely related species, the prevalent model in the field is that CRMs operating in these species are functionally equivalent, as has been shown for some test cases [12]. However, a recent study found patterns of variation in Drosophila blastoderm CRMs that are inconsistent with a nearly neutral process [48]. As Kreitman and colleagues point out in that paper, “the assumption of CRM functional stasis, which is the main argument for the neutral (i.e., compensatory) view is not well supported experimentally.” Though not attributable to differences in CRMs without further study, we do provide evidence of quantitative differences in expression for many genes in the segmentation network between closely related Drosophila species.

Alternatively, the proportion of cell types may be selected upon directly, as they could contribute to organismal phenotypes by propagating through later stages of development to create fine-scale differences between these species. This would represent selection on a quantitative intermediate developmental trait, likely mediated by the type of small scale differences in expression for multiple genes we observe in our dataset. This scenario would differ from selection on macroscopic terminal organismal phenotypes such as changes in pigmentation, bristle number and skeletal structures, where small numbers of loci or even single loci, of large effect have been identified [49]–[52].

Finally, it is possible that the differences in gene expression are a consequence of selection on egg size and morphology. Egg size is known to be a selectable trait and to vary significantly across populations [53]–[55]. The expression differences we see would then reflect how the segmentation network has been fine-tuned to operate in different morphological contexts while maintaining the proper allocation of cell types. This idea was also recently put forth by Kreitman and colleagues to account for evidence of positive selection on Drosophila blastoderm CRMs, as mentioned above [48]. They term this the “moving target” hypothesis, and posit that input functions must constantly adapt to changing conditions within the embryo. We favor this hypothesis as well. It remains a future challenge to identify both the target of selection for this network, and the design principles that confer its robustness to genetic and morphological perturbation.

## Materials and Methods

### Embryo collection and fixation

Embryos were collected, fixed and prehybridized according to standard protocols, which are available at http://depace.med.harvard.edu/links.html, and described in [6], [26]. Briefly, *D. yakuba* and *D. pseudoobscura* cages were maintained at 23°C. *D. yakuba* embryos were collected for 3 hours, and aged for 2 hours prior to fixation. *D. pseudoobscura* embryos were collected for 3 hours, and aged for 3 hours prior to fixation. Embryos were dechorionated in 50% bleach for 3 minutes, washed, and fixed in a 1∶4 solution of 10% formaldehyde (Polysciences #04018) to heptane for 20 minutes with vigorous shaking. The vitelline membranes were removed by shaking with MeOH and washed 3X with 100% MeOH. Fixed embryos were stored at −20°C in 100% ethanol. Embryos were pooled for prehybrization, rehydrated in PBT + Tx (PBS pH 7.2, 0.05% Tween20 and 0.2% Triton X-100), post-fixed for 20 minutes in 5% formaldehyde in PBT+Tx, washed in hybridization buffer (50% formamide, 5X SSC pH 5.2, 0.2% Triton X-100, 40 µg/ml heparin, and 250 µg/ml salmon sperm DNA) and incubated at 55°C for 1 to 5 hours in hybridization buffer. Prehybridized embryos were stored in hybridization buffer at −20°C.

### Probe synthesis and *in situ* hybridization

There were two modifications to the staining protocol developed for *D. melanogaster* [6]. First, species-specific RNA probes were made using cDNA or genomic DNA as a template, whereas cDNA probes were used exclusively for the *D. melanogaster* data. Probes ranged in size from 531 bp to 2771 bp, and either encompassed the majority of the coding sequence or overlapped large exons (Table S4). Variation in probe length did not significantly affect our measurements (Figure S11). Second, two different haptens are required for our imaging pipeline, one for the registration gene and one for the gene of interest. While dinitrophenol (DNP) - labeled probes gave consistently clean results in all species, digoxygenin (DIG) - labeled probes yielded variable levels of background. Another commonly used hapten, biotin, was even worse. Because DIG stains were strong enough to reliably distinguish stripes, we chose to use it for the registration channel, but not include the data in the final gene expression atlases.

Probe templates were cloned by PCR amplification using either genomic DNA or cDNA libraries as a template, ligated into pGEM-Teasy, and sequence verified. Cloning primers are listed in Table S4. Probe templates were generated by PCR with M13 forward and reverse primers. Anti-sense digoxygenin (DIG) or dinitrophenol (DNP) probes were synthesized using in vitro transcription from DNA templates using either SP6 or T7 polymerase, depending on the orientation of the clone. Probes were not carbonate-treated as this did not improve stain quality. All probes were diluted to 200 ng/µl.

For *in situ* hybridizations, approximately 100 µl of embryos were incubated for up to 48 hours at 55–57°C in 300 µl of hybridization buffer with 2–10 µl each of a DIG and DNP probe. Embryos were then washed extensively with hybridization buffer at 55–57°C, and probes were detected sequentially using horseradish-peroxidase (HRP) conjugated antibodies (anti-DIG POD, Roche 11207733910 at 1∶250 or 1∶500; anti-DNP Perkin Elmer NEL747 A001KT at 1∶100) and either coumarin or Cy3 tyramide amplification (Perkin-Elmer NEL703 001KT, SAT 704B). To disable the HRP in the first signal detection reaction, embryos were washed in hybridization buffer at 55°C and incubated in 5% formaldehyde in PBT+Tx for 20 minutes. All remaining RNA was removed by incubation with 0.18 µg/ml RNAse A in 500 µl PBT+Tx overnight at 37°C. Nuclei were detected by staining with Sytox Green (Molecular Probes #S7020, 1∶5000 in 500 µl overnight at 4°C). Embryos were dehydrated in an ethanol series and mounted in xylene-based DePex (Electron Microscopy Service #13514) on a slide with 2 bridging coverslips to prevent flattening of the embryos. Detailed protocols are available at http://depace.med.harvard.edu/links.html.

### Image acquisition, analysis, and registration

Three-dimensional image stacks of individual embryos were acquired semi-automatically on a Zeiss LSM 710 using a plan-apochromat 20X 0.8NA objective. Embryos were located, staged using phase contrast optics, and the imaging parameters such as the height of the image stack and gain settings for each fluorophore were recorded. A custom built macro then acquired all marked embryos [7]. All three fluorophores (Sytox Green, coumarin and Cy3) were excited simultaneously at 750 nm, using a Coherent Chameleon 2-photon laser at 4–7% power. The emission was spectrally split into 3 channels: 462–502 nm (coumarin), 514–543 nm (sytox), 599–676 nm (Cy3). Images were 1024×1024, and slices were taken every 1 µm. Resulting image stacks were processed by previously described algorithms to unmix channels [56], and segment individual nuclei [6], resulting in individual pointcloud files for each embryo. These were housed in a custom-built database.

Gene expression atlases were assembled using the registration algorithms previously described in [7]. For each species, a morphological model was constructed that contained an average number of nuclei. The 3D positions of the nuclei in the model were chosen to match the average egg-length, shape and density pattern measured for each of the 6 temporal cohorts. Motions of nuclei between time points in the model were constrained to be as small and smooth as possible while still recapitulating the observed changes in density and shape (see [27] for details). Pointcloud data extracted for each embryo in a given cohort were aligned to the morphological template by a rigid-body transformation and isotropic scaling. For each time point, a registration template was constructed by finding average boundary locations of a registration marker gene (eve or ftz) with respect to the egg-length of the morphological model. Fine registration of individual embryo pointclouds was then carried out by non-rigid warping of the embryo to align marker gene boundaries with the template. Finally, expression values were computed for each nucleus and time point in the model by averaging measurements across those nuclei in individual pointclouds that were closest after spatial registration. Prior to averaging, gains and offsets were estimated for expression measurements within each embryo pointcloud in order to minimize the expression variance across the cohort and to match smoothed estimates of the total change in expression level between temporal cohorts (see [7] for details).

### Calculation of surface area and density

Surface area was computed as the sum of areas of the triangles defined by the neighbor relation information in the Pointclouds [6]. Local density was computed by defining a disk of 15 µm radius on the surface around each nucleus, and dividing the number of nuclei in this disk by its area [6]. These density maps were then averaged over a cohort of embryos by resampling the cylindrical projections onto a regular grid.

### Calculation of expression distance score

Cell-to-cell comparisons within and between species were made by looking at the squared distance between vectors of average expression measurements for the cell at all 6 time points and 11 genes. For a pair of nuclei ***i*** and ***j*** we computed the distance:
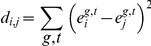
where **e**_i_^gt^ is the expression of the ***g***th gene recorded in the atlas for the ***i***th cell at time point ***t***. We used squared distance since it is additive across genes and time-points which makes the contribution of individual genes more interpretable. Prior to computing the distance, expression levels for each gene in the atlas were scaled so that the maximum expression at each time point was 1.0. In order to determine relevant cells to compare between species, only cells that were nearby were considered. Corresponding locations were estimated by scaling each atlas to unit egg length and nearby nuclei were specified as those nuclei in the target embryo that were within the 30 nearest to the cell to be matched.

Since there are often several cells that are good matches, the displacement direction to the best matching nearby cell is noisy. In order to visualize displacement, we used a weighted average of the locations of the top 10 matching cells (smallest expression distance). The 3D locations of these 10 matching cells were averaged using weights inversely proportional to the expression distance (i.e. 1/***d_ij_***). These 3D displacement vectors were then visualized on a cylindrical projection.

We chose not to include bcd and cad in calculation of the expression distance score for the entire dataset. We excluded bcd because its expression increases over the first two time points in the *D. melanogaster* dataset; this is likely an experimental artifact and leads to artificially high expression distance scores in the anterior. We excluded cad because data was not available for *D. pseudoobscura* and we wished to compare results between the *D. melanogaster/D. yakuba* and *D. melanogaster/D. pseudoobscura* analyses.

### Calculating statistical significance for expression differences

As an alternative to expression distance scores, we also considered a hypothesis-testing framework in which two cells are declared to have different expression profiles if the expression of some gene at some time point is significantly different relative to variance in our measurements. This comparison was carried out independently for each cell, gene and time-point using a two-sample t-test with unequal sample sizes and variances. In all tests we used the Bonferroni correction to assure a family-wise error rate of less than 0.01. Visualizations and histograms in Figures S5 and S9 show the number of expression profile measurements for which a given cell was significantly different under this significance threshold.

### Calculating relative proportions of cell types

We defined an expression neighborhood **N_i_** for a given nucleus **i** in the following way. Choose a threshold **t** and find all nuclei in the atlas for which **d_ij_**<**t**. Of these nuclei with similar spatio-temporal expression profiles, let **N_i_** be the largest connected component on the embryo surface that contains cell **i**. For areas where the expression pattern varies rapidly in space, this neighborhood of similar cells is small. In areas where the pattern changes slowly, the neighborhood is large.

To determine how these neighborhoods might expand or contract between different species, we consider the neighborhood size around corresponding nuclei. Let ***j*** be the nucleus in the target atlas whose expression profile best matches ***i*** in the source atlas. We compare the relative sizes of the two neighborhoods in order to gauge the degree of expansion or contraction measured by the log ratio of neighborhood sizes:
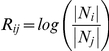



The log ratio is symmetric about zero with positive values indicating an expansion and negative values indicating a contraction.

One concern is that the choice of neighbor threshold may affect this analysis since there is not a meaningful way to scale the measured fluorescence levels between atlases of different species. To resolve this, we choose the threshold for each target atlas adaptively. Given a fixed threshold for the query atlas, we searched over thresholds for the target atlas in order to find a threshold in which the average expansion ratio R across all cells matched the log-ratio of the number of nuclei in the two atlases. Choosing the threshold in this way entails that ratios are visualized relative to a null hypothesis of uniform scaling between species.

### Visualization of expression distance scores and underlying cellular gene expression profiles using MulteeSum

Figures similar to Figure 5, Figure 6, Figure S4, Figure S7, and Figure S8 can easily be generated using MulteeSum, a custom software tool for visualizing comparative analysis of cellular gene expression profiles [57]. Our datasets are complex and best viewed interactively. We therefore have made MulteeSum and the analyses presented here available for download at http://depace.med.harvard.edu/downloads/MulteeSum.zip. We have released MulteeSum open source, and it was developed using the Processing programming language (http://www.processing.org), an open-source language for visualization. Executables for running on Mac OSX, Windows and Linux and instructions (see README.txt) are included in the download. A full description of the usage and features of MulteeSum can be found at http://www.multeesum.org.

## Supporting Information

Figure S1 *D. melanogaster*, *D. yakuba*, and *D. pseudoobscura* atlases are of similar quality. The frequencies of relative intensities for each gene in each atlas are compared (*D. melanogaster* in blue, *D. yakuba* in orange and *D. pseudoobscura* in green). For stains with obvious non-specific background, the peak of the distribution becomes quite broad.
(TIF)

Figure S2 The density patterns of *D. melanogaster*, *D. yakuba*, and *D. pseudoobscura* embryos are statistically distinct. Point-wise nuclei density estimates for each cohort were compared between species using a paired t-test. Plots show the log p-values for this comparison. With the exception of the white areas, the differences in densities are statically significant (p≥0.05). The density of nuclei in *D. melanogaster* and *D. yakuba* are different from one another with high statistical significance, likewise for the density patterns of *D. yakuba* and *D. pseudoobscura*, with the exception of a small anterior dorsal region in the early time point, representing only 1% of nuclei. The greatest similarity in density levels are corresponding areas of *D. melanogaster* and *D. pseudoobscura* constituting roughly 15, 11 and 33 percent of the nuclei during the early, middle and late time points respectively. Anterior to the left, D dorsal, L lateral, V ventral.
(TIF)

Figure S3 Cell flow models in *D. melanogaster*, *D. yakuba* and *D. pseudoobscura* atlases are similar. Panels show the estimated movements of nuclei based on the average shape and density of imaged embryos in each temporal cohort using the method described in [24]. Lines show the direction of motion. Since the cylindrical projection distorts distances near the poles, the color of each line indicates the distance in 3D as a proportion of egg length. Despite differences in density patterns (see Figure S2), the estimated cell flow is quite similar across all three species. As noted in the main paper, this flow is incorporated into the atlas and hence automatically factored out of our comparative expression analysis.
(TIF)

Figure S4 Individual gene expression patterns vary in relative position and intensity. (1st row) For each *D. melanogaster* query cell, the expression distance score of the nearest target cell in *D. yakuba* (left) and *D. pseudoobscura* (right) is shown. (2nd row) The expression distance score for the best-matched cell within the nearest 30 for both *D. yakuba* and *D. pseudoobscura* is shown. High expression distance scores, indicating poor matches, are darker. All cells scoring above 0.7 are colored the darkest blue; the maximum value amongst all cells is reported at the top of the color map. (3rd row) For each *D. melanogaster* query cell, the distance and direction to the average position of the top 10 best corresponding target cells is shown. The correspondence is shown with a line that starts at the position of the query cell, and ends at the average position of the target cells. The end of the line is indicated with a black dot. Because the 2D projection distorts actual distance in 3D, the lines are color-coded to indicate actual distance traversed in 3D. Blue is a large distance, yellow is a small distance. (4th row) The distribution of expression distance scores using only the nearest cell (grey) and best-matched cell within the nearest 30 (blue) are shown. The distribution of scores narrows and the mode decreases after a local search. To establish the significance of the calculated differences, we assembled two atlases from the *D. melanogaster* dataset, and compared these two atlases to each other (dotted lines). We show data from fkh, ftz, gt, hb, hkb, kni, Kr, odd, prd and tll, which together with eve (described in Figure 5) form the set of 11 genes used for analyzing the whole gene expression profile.
(PDF)

Figure S5 Even after local searching, some cells have statistically different even-skipped gene expression profiles. As an alternative to the expression distance score (Figure 5), we evaluate the similarity of matched cells using independent pair-wise comparisons for eve expression at each time point. Top panels show the number of time points for which the measured expression level of eve in the corresponding cell was significantly different. (1st row) For each *D. melanogaster* query cell, the number of significant expression differences with the nearest target cell in *D. yakuba* (left) and *D. pseudoobscura* (right) is shown. (2nd row) The number of significant expression differences score for the best matched cell within the nearest 30 for both *D. yakuba* and *D. pseudoobscura* is shown. Numbers above each panel indicate the number of cells which were significantly different at one or more time points at a family-wise error rate (FWER) of 0.01. Histograms at bottom show the number of cells whose expression profile differed significantly at a given number of entries (total number of entries  = no. of genes x no. of time points in expression profile). Histograms also show a control that compared different *D. mel* atlases constructed from two disjoint sets of embryos. Expression levels in the paired control atlases are statistically identical at this confidence level for nearly all nuclei.
(TIF)

Figure S6 The boundaries of even-skipped expression are in different relative positions between *D. melanogaster*, *D. yakuba*, and *D. pseudoobscura*. Individual pointclouds were divided into 16 dorsal-ventral strips, and the position of the boundaries of eve expression in each strip was measured as in [6]. The average position and 95% confidence intervals at each of the 16 positions are plotted for embryos early in cellularization (4–25%) and later in cellularization (51–100%).
(TIF)

Figure S7 When comparing even-skipped expression, unmatched cells in *D. yakuba* and *D. pseudoobscura* are rare. Because we do not require a one-to-one match, there are potentially cells in the target embryos without any matches. We tallied matches for all target cells by awarding 1 count if the cell appeared in the top 10 hits for a given query cell. The expression distance score is sensitive to even small differences in expression profiles; tallying the top 10 distinguishes between target cells that are unmatchable due to more extreme expression differences from those that just aren't quite perfect. The number of matches for each cell in the target embryos is shown. The color map was binned into 6 populations according to the distribution of matches for each target embryo. Unmatched cells (dark brown) are almost exclusively found outside the area of even-skipped expression. The few unmatched cells within the area of even-skipped expression are intermingled with more highly matched cells, and are only subtly different from their neighbors (expression profiles can be viewed in MulteeSum (see Materials and Methods) by clicking on these cells).
(TIF)

Figure S8 When comparing the whole gene expression profile (hb, gt, Kr, kni, fkh, hkb, tll, eve, ftz, odd, and prd), unmatched cells in *D. yakuba* and *D. pseudoobscura* are rare and similar to their matched neighbors. If there were cells that had substantially different expression profiles, such as complete lack of expression of a certain gene, they may be avoided by our matching protocol. To assess this possibility, we tallied matches for all target cells by awarding 1 count if the cell appeared in the top 10 hits for a given query cell. The number of matches for each cell in the target embryos is shown. The color map was binned into 6 populations according to the distribution of matches for each target embryo. A few unmatched cells (dark brown) are found at the poles and are intermingled with more highly matched cells; these cells are only subtly different from their neighbors (expression profiles can be viewed in MulteeSum (see Materials and Methods) by clicking on these cells).
(TIF)

Figure S9 Even after local searching, some cells have statistically different gene expression profiles. As an alternative to the expression distance score (Figure 6), we evaluate the similarity of matched cells using independent pair-wise comparisons for all 11 gene expression levels at each time point. Top panels show the number of expression profile entries for which the measured expression level of in the corresponding cell was significantly different. (1st row) For each *D. melanogaster* query cell, the number of significant expression differences with the nearest target cell in *D. yakuba* (left) and *D. pseudoobscura* (right) is shown. (2nd row) The number of significant expression differences score for the best matched cell (smallest expression distance) within the nearest 30 for both *D. yakuba* and *D. pseudoobscura* is shown. Numbers above each panel indicate the number of cells that were significantly different at one or more profile entries at a family-wise error rate (FWER) of 0.01. Histograms at bottom show the number cells whose expression profile differed significantly at a given number of entries (genes/time points). Histograms also show a control that compared different *D. mel* atlases constructed from two disjoint sets of embryos. Expression levels in the paired control atlases are statistically identical at this confidence level for nearly all nuclei. Even after local searching, some cells have statistically different gene expression profiles. As an alternative to the expression distance score (Figure 6), we evaluate the similarity of matched cells using independent pair-wise comparisons for all 11 gene expression levels at each time point. Top panels show the number of expression profile entries for which the measured expression level of in the corresponding cell was significantly different. (1st row) For each *D. melanogaster* query cell, the number of significant expression differences with the nearest target cell in *D. yakuba* (left) and *D. pseudoobscura* (right) is shown. (2nd row) The number of significant expression differences score for the best matched cell (smallest expression distance) within the nearest 30 for both *D. yakuba* and *D. pseudoobscura* is shown. Numbers above each panel indicate the number of cells that were significantly different at one or more profile entries at a family-wise error rate (FWER) of 0.01. Histograms at bottom show the number cells whose expression profile differed significantly at a given number of entries (genes/time points). Histograms also show a control that compared different *D. mel* atlases constructed from two disjoint sets of embryos. Expression levels in the paired control atlases are statistically identical at this confidence level for nearly all nuclei.
(TIF)

Figure S10 Expression differences are widespread throughout the network. Corresponding cells were identified by searching amongst the nearest 30 cells and scoring the whole gene expression profile (hb, gt, Kr, kni, fkh, hkb, tll, eve, ftz, odd, prd). The contributions to the expression distance score for the best-matched cell were calculated for various tiers of the network (the gap and terminal genes, the pair-rule genes as a group and the individual pair-rule genes). High expression distance scores, indicating poor matches, are darker. All cells scoring above 1.0 are colored the darkest blue; when the maximum value exceeds 1.0, the maximum value amongst all cells is reported at the top of the color map.
(TIF)

Figure S11 Probe length does not significantly effect position measurements. ftz expression was measured using either a 988 bp exonic (dark green) or a 1350 bp cDNA (light green) *in situ* probe. Individual pointclouds were divided into 16 dorsal/ventral strips, and the position of the boundaries of ftz expression in each strip was measured. The average position and 95% confidence intervals at each of the 16 positions are plotted for embryos later in cellularization (51–100%).
(TIF)

Table S1 Number of embryos per cohort in *D. yakuba* and *D. pseudoobscura* datasets. The number of embryos per gene varies across the dataset with a minimum of 4 embryos per gene per time point. Data was not collected for time points when the gene is not expressed (i.e. Bcd at later time points). We collected additional data on the registration genes eve and ftz in both species.
(DOC)

Table S2 Standard deviation of gene expression is reduced after registration to similar levels in *D. melanogaster*, *D. yakuba*, and *D. pseudoobscura* atlases. The average standard deviation after registration is shown for all genes in the D. yakuba and D. pseudoobscura atlases, as well as the corresponding data from the *D. melanogaster* atlas for comparison [7].
(DOC)

Table S3 Mean and median expression distance score before and after local search. A local search significantly decreases the expression distance score relative to direct spatial mapping. The mean and median of the expression distance score using direct spatial mapping (nearest cell) and local search (local search) is shown for pair-wise comparisons between *D. melanogaster* and *D. yakuba* and *D. melanogaster* and *D. pseudoobscura*, for both individual genes and all genes in our dataset.
(DOC)

Table S4 Probe constructs used for *D. yakuba* and *D. pseudoobscura in situ* hybridizations. The gene, species, template type (cDNA or genomic DNA (gDNA), length, and primers are shown for each in situ probe used in this study.
(DOC)
