# Supplementary figures and images for: A Conserved Developmental Patterning Network Produces Quantitatively Different Output in Multiple Species of Drosophila
Source: PLoS Genet. 2011 Oct 27;7(10):e1002346. doi: 10.1371/journal.pgen.1002346 (PMC3203197; doi:10.1371/journal.pgen.1002346)

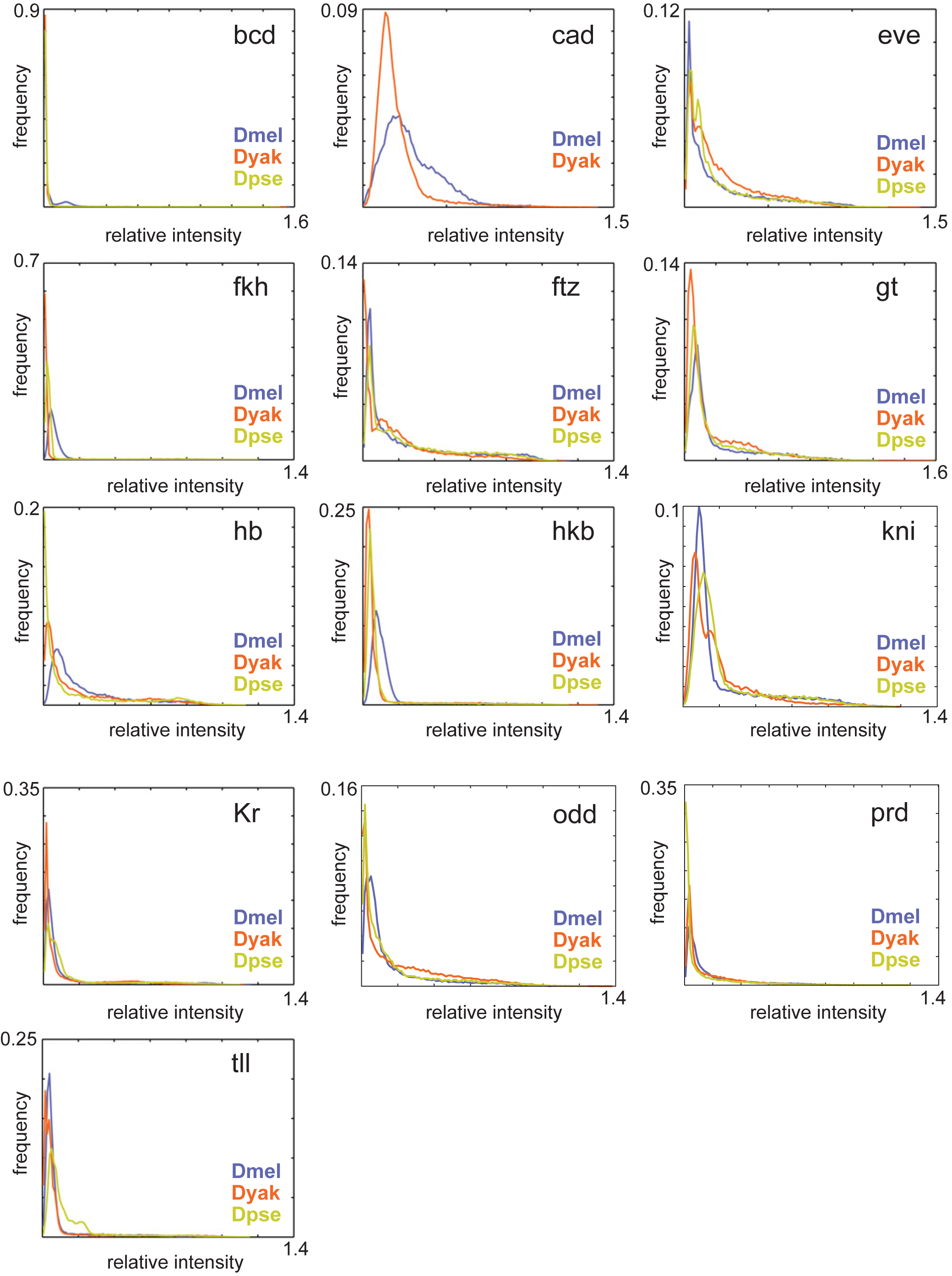

Supplement: Figure S1 — D. melanogaster, D. yakuba, and D. pseudoobscura atlases are of similar quality. The frequencies of relative intensities for each gene in each atlas are compared (D. melanogaster in blue, D. yakuba in orange and D. pseudoobscura in green). For stains with obvious non-specific background, the peak of the distribution becomes quite broad. (TIF) [file pgen.1002346.s001.tif]

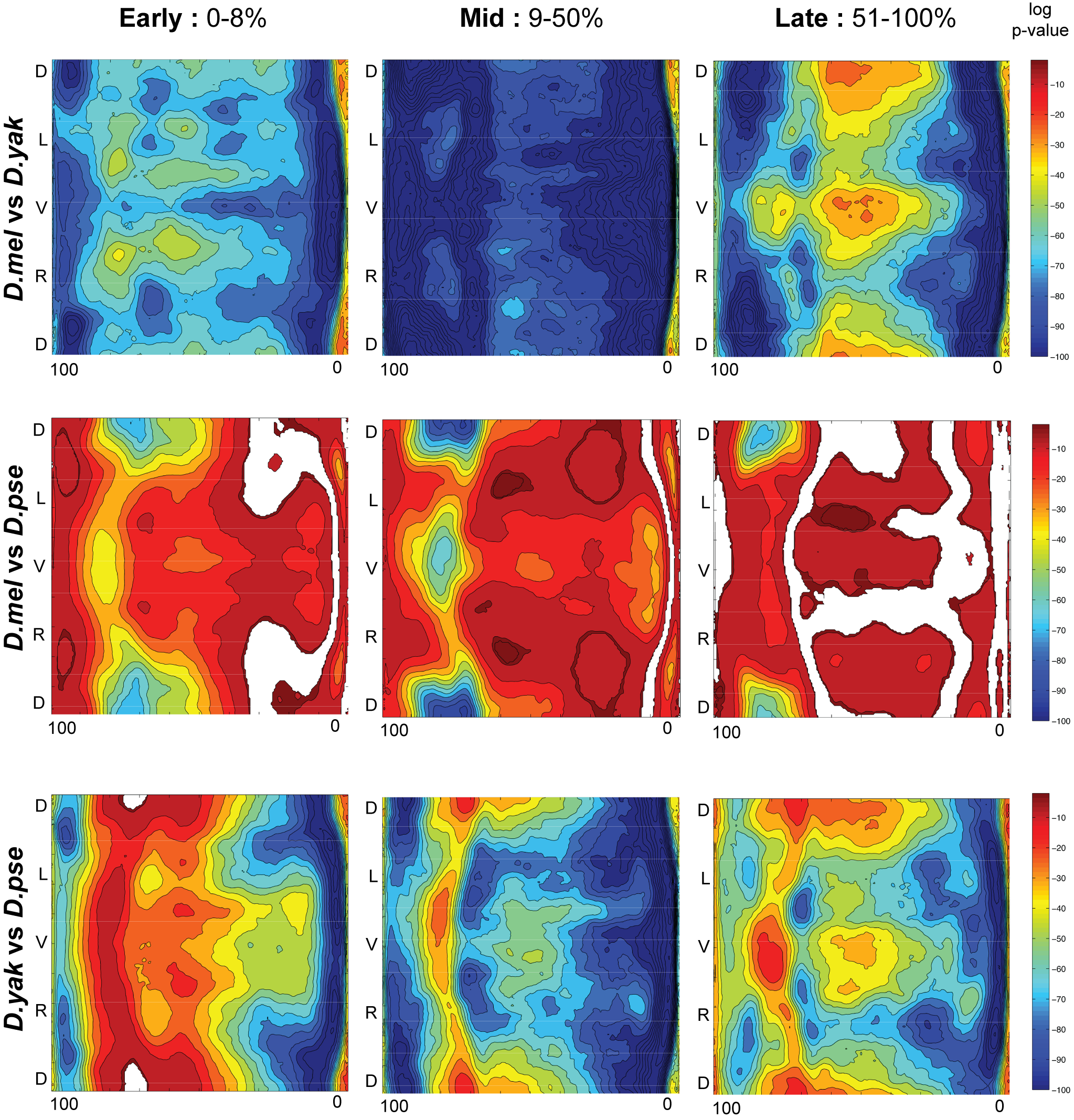

Supplement: Figure S2 — The density patterns of D. melanogaster, D. yakuba, and D. pseudoobscura embryos are statistically distinct. Point-wise nuclei density estimates for each cohort were compared between species using a paired t-test. Plots show the log p-values for this comparison. With the exception of the white areas, the differences in densities are statically significant (p≥0.05). The density of nuclei in D. melanogaster and D. yakuba are different from one another with high statistical significance, likewise for the density patterns of D. yakuba and D. pseudoobscura, with the exception of a small anterior dorsal region in the early time point, representing only 1% of nuclei. The greatest similarity in density levels are corresponding areas of D. melanogaster and D. pseudoobscura constituting roughly 15, 11 and 33 percent of the nuclei during the early, middle and late time points respectively. Anterior to the left, D dorsal, L lateral, V ventral. (TIF) [file pgen.1002346.s002.tif]

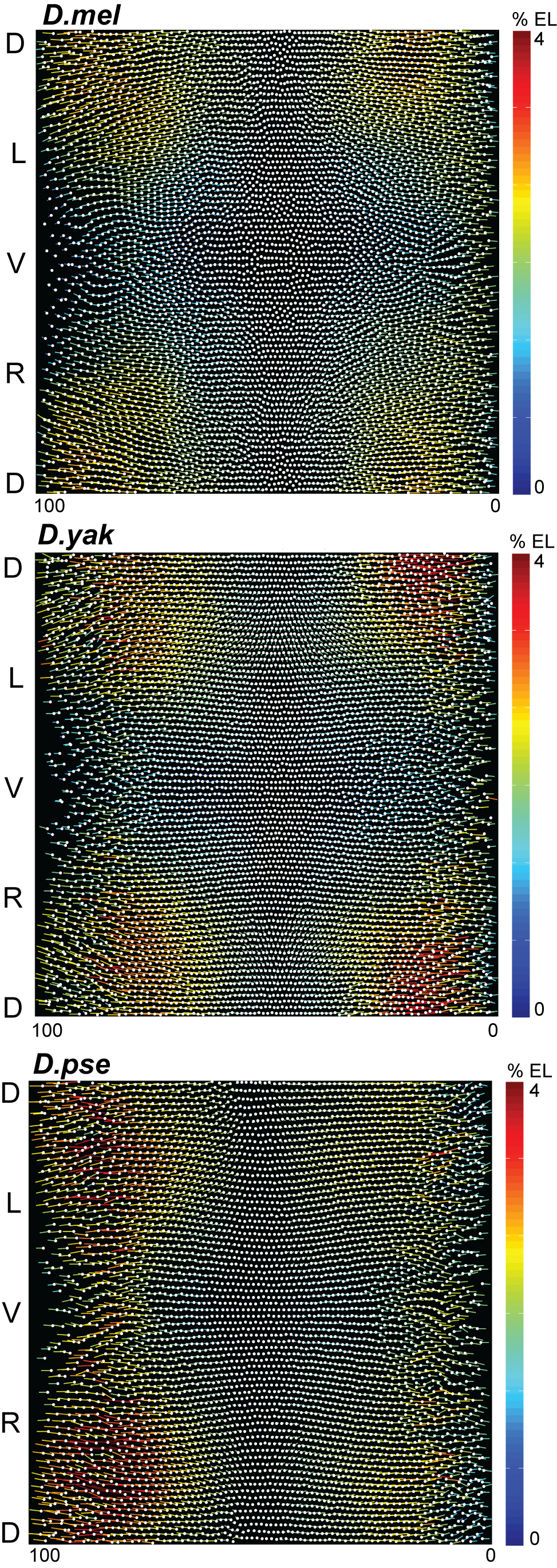

Supplement: Figure S3 — Cell flow models in D. melanogaster, D. yakuba and D. pseudoobscura atlases are similar. Panels show the estimated movements of nuclei based on the average shape and density of imaged embryos in each temporal cohort using the method described in [24]. Lines show the direction of motion. Since the cylindrical projection distorts distances near the poles, the color of each line indicates the distance in 3D as a proportion of egg length. Despite differences in density patterns (see Figure S2), the estimated cell flow is quite similar across all three species. As noted in the main paper, this flow is incorporated into the atlas and hence automatically factored out of our comparative expression analysis. (TIF) [file pgen.1002346.s003.tif]

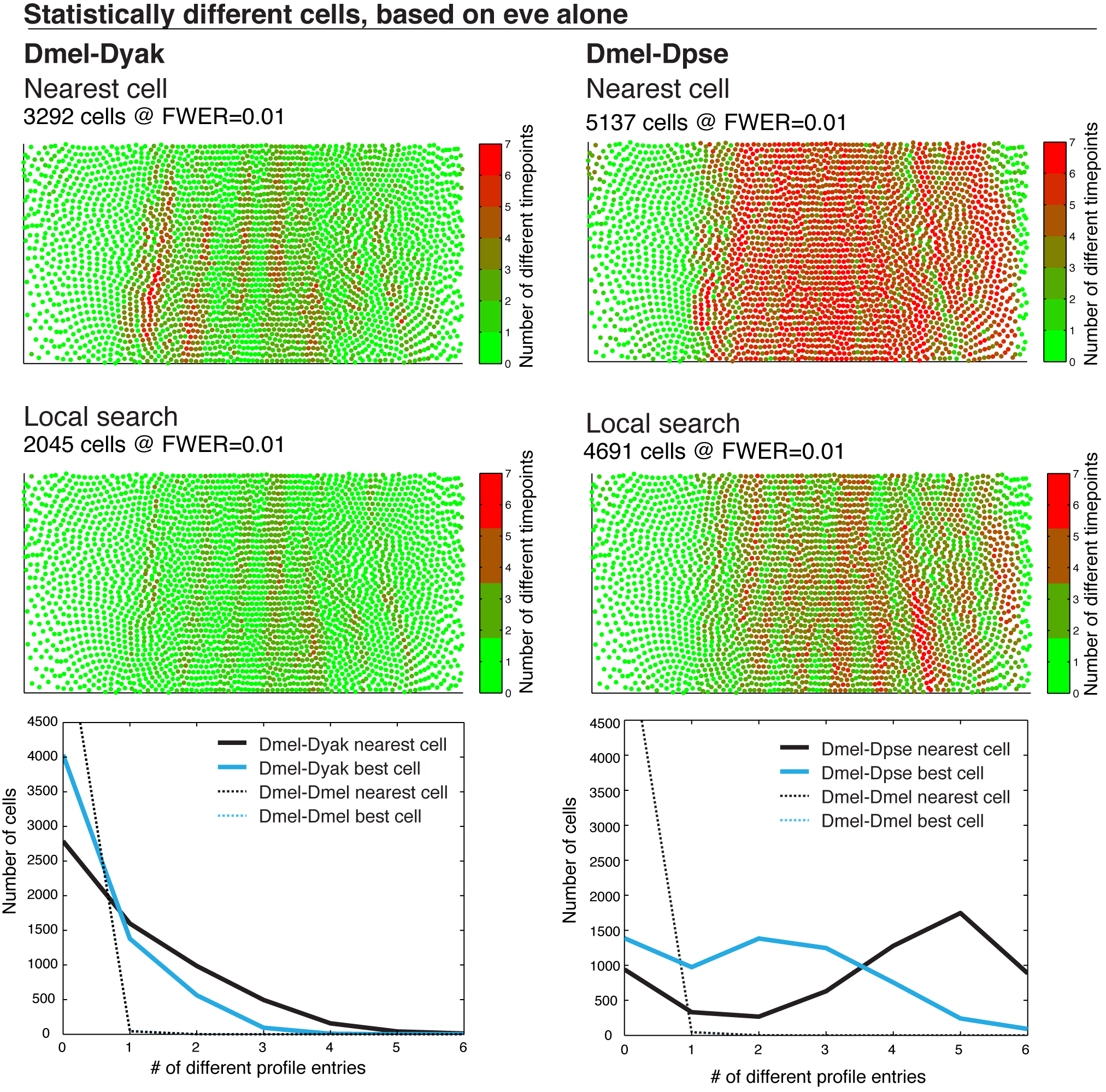

Supplement: Figure S5 — Even after local searching, some cells have statistically different even-skipped gene expression profiles. As an alternative to the expression distance score (Figure 5), we evaluate the similarity of matched cells using independent pair-wise comparisons for eve expression at each time point. Top panels show the number of time points for which the measured expression level of eve in the corresponding cell was significantly different. (1st row) For each D. melanogaster query cell, the number of significant expression differences with the nearest target cell in D. yakuba (left) and D. pseudoobscura (right) is shown. (2nd row) The number of significant expression differences score for the best matched cell within the nearest 30 for both D. yakuba and D. pseudoobscura is shown. Numbers above each panel indicate the number of cells which were significantly different at one or more time points at a family-wise error rate (FWER) of 0.01. Histograms at bottom show the number of cells whose expression profile differed significantly at a given number of entries (total number of entries = no. of genes x no. of time points in expression profile). Histograms also show a control that compared different D. mel atlases constructed from two disjoint sets of embryos. Expression levels in the paired control atlases are statistically identical at this confidence level for nearly all nuclei. (TIF) [file pgen.1002346.s005.tif]

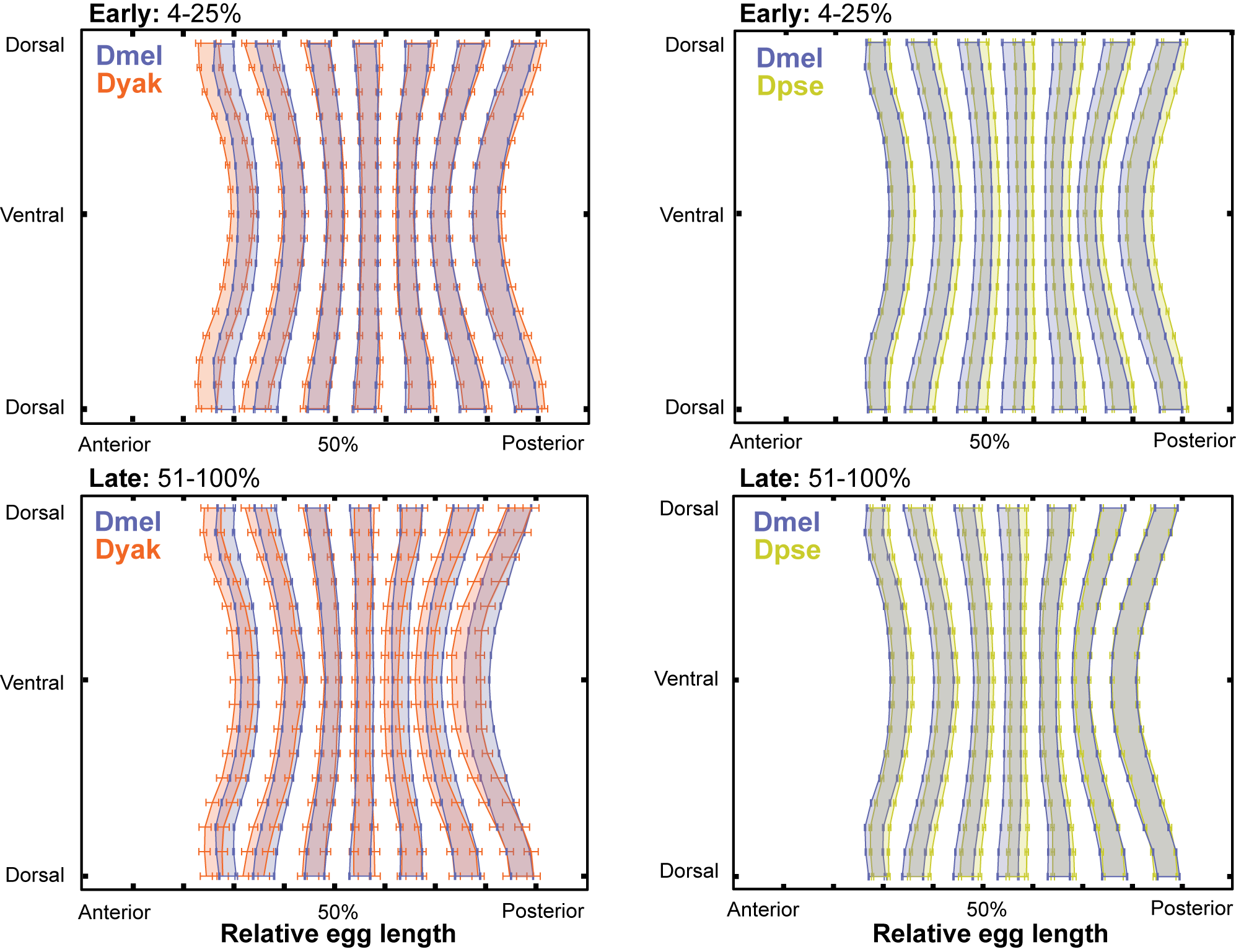

Supplement: Figure S6 — The boundaries of even-skipped expression are in different relative positions between D. melanogaster, D. yakuba, and D. pseudoobscura. Individual pointclouds were divided into 16 dorsal-ventral strips, and the position of the boundaries of eve expression in each strip was measured as in [6]. The average position and 95% confidence intervals at each of the 16 positions are plotted for embryos early in cellularization (4–25%) and later in cellularization (51–100%). (TIF) [file pgen.1002346.s006.tif]

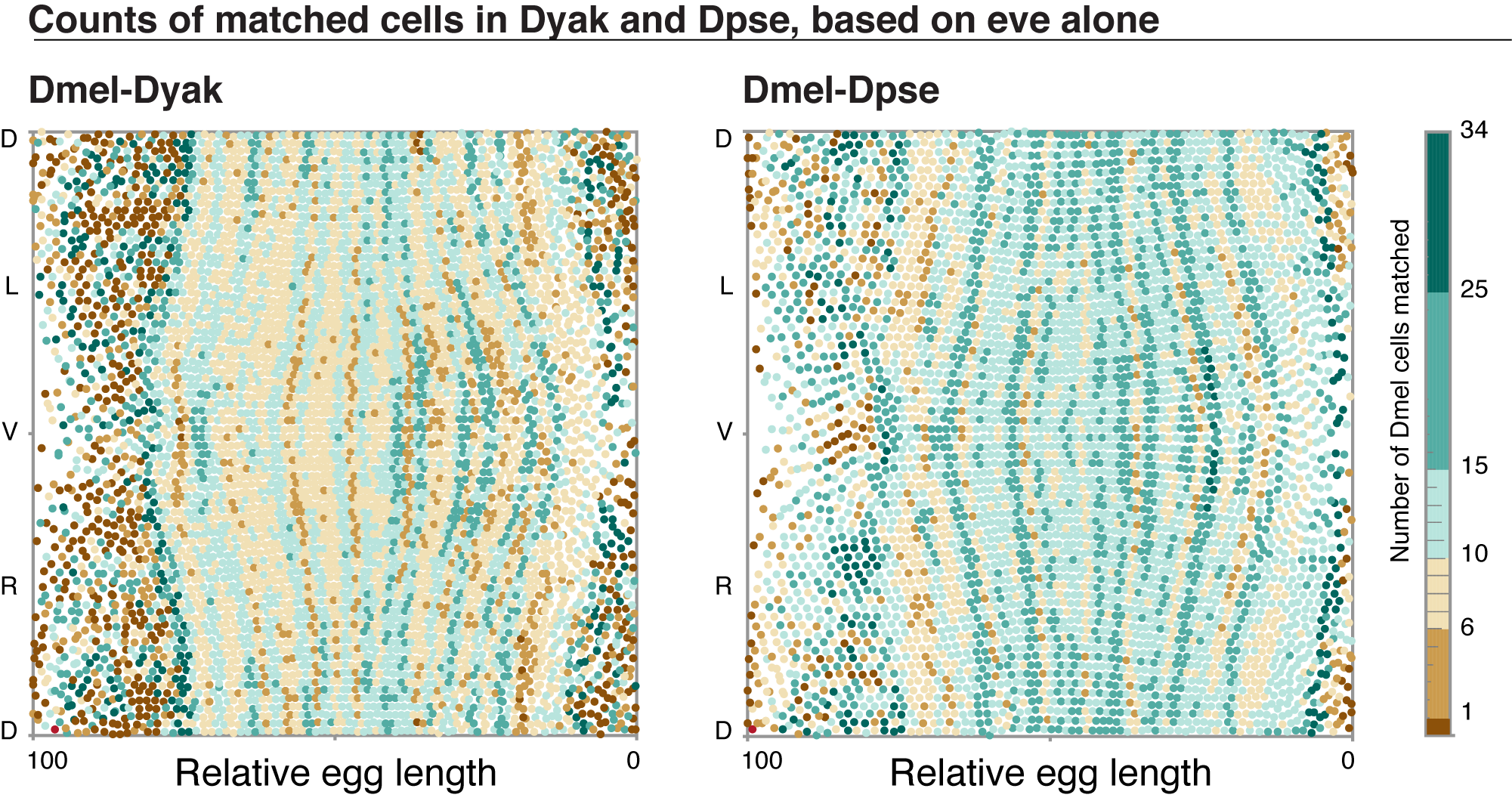

Supplement: Figure S7 — When comparing even-skipped expression, unmatched cells in D. yakuba and D. pseudoobscura are rare. Because we do not require a one-to-one match, there are potentially cells in the target embryos without any matches. We tallied matches for all target cells by awarding 1 count if the cell appeared in the top 10 hits for a given query cell. The expression distance score is sensitive to even small differences in expression profiles; tallying the top 10 distinguishes between target cells that are unmatchable due to more extreme expression differences from those that just aren't quite perfect. The number of matches for each cell in the target embryos is shown. The color map was binned into 6 populations according to the distribution of matches for each target embryo. Unmatched cells (dark brown) are almost exclusively found outside the area of even-skipped expression. The few unmatched cells within the area of even-skipped expression are intermingled with more highly matched cells, and are only subtly different from their neighbors (expression profiles can be viewed in MulteeSum (see Materials and Methods) by clicking on these cells). (TIF) [file pgen.1002346.s007.tif]

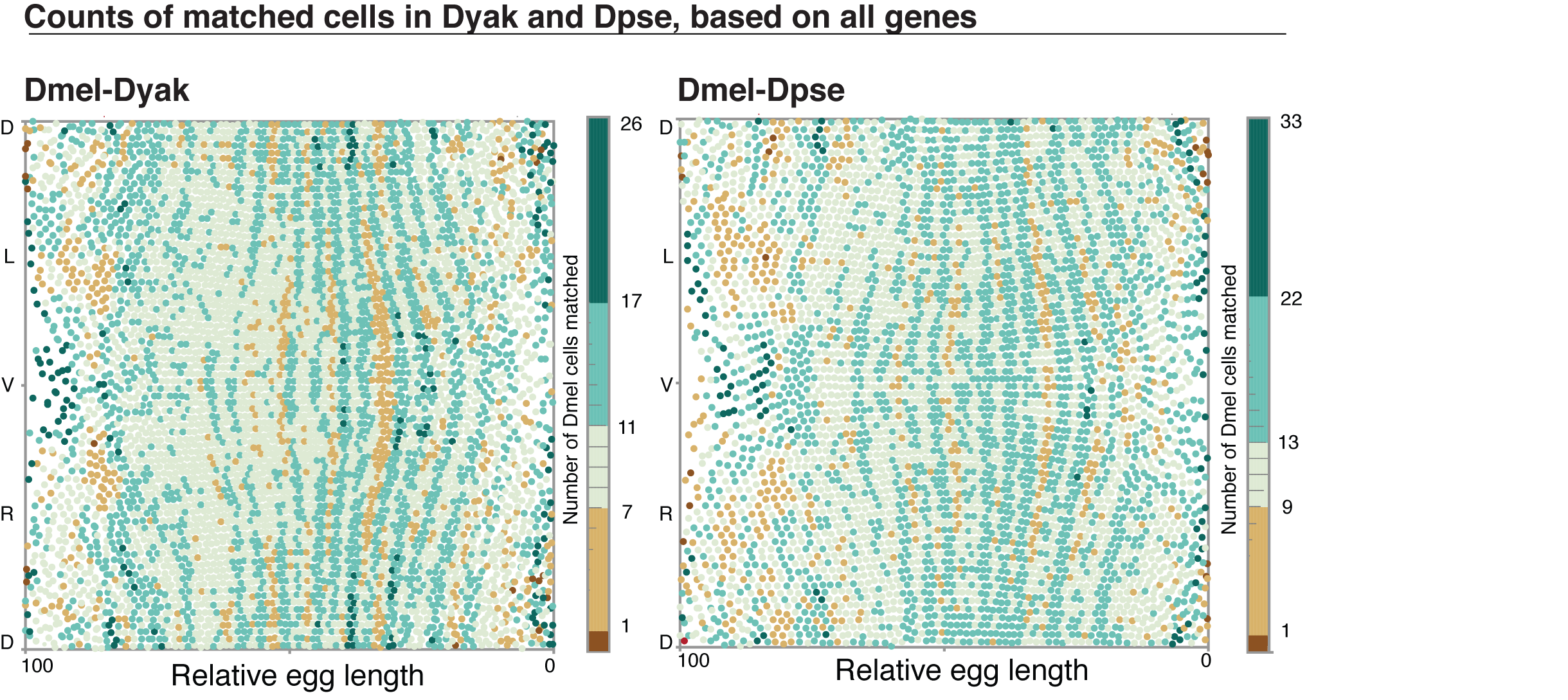

Supplement: Figure S8 — When comparing the whole gene expression profile (hb, gt, Kr, kni, fkh, hkb, tll, eve, ftz, odd, and prd), unmatched cells in D. yakuba and D. pseudoobscura are rare and similar to their matched neighbors. If there were cells that had substantially different expression profiles, such as complete lack of expression of a certain gene, they may be avoided by our matching protocol. To assess this possibility, we tallied matches for all target cells by awarding 1 count if the cell appeared in the top 10 hits for a given query cell. The number of matches for each cell in the target embryos is shown. The color map was binned into 6 populations according to the distribution of matches for each target embryo. A few unmatched cells (dark brown) are found at the poles and are intermingled with more highly matched cells; these cells are only subtly different from their neighbors (expression profiles can be viewed in MulteeSum (see Materials and Methods) by clicking on these cells). (TIF) [file pgen.1002346.s008.tif]

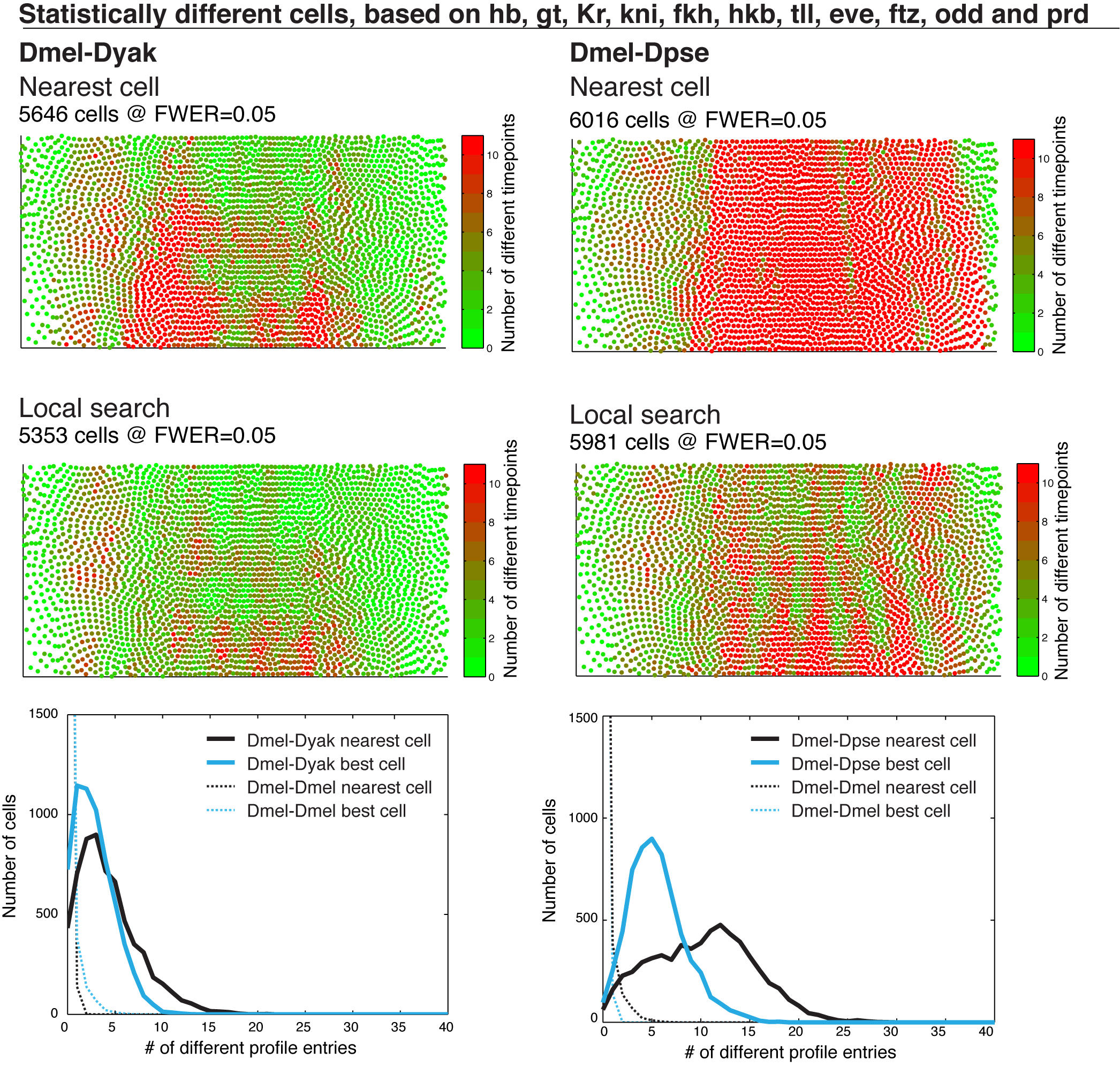

Supplement: Figure S9 — Even after local searching, some cells have statistically different gene expression profiles. As an alternative to the expression distance score (Figure 6), we evaluate the similarity of matched cells using independent pair-wise comparisons for all 11 gene expression levels at each time point. Top panels show the number of expression profile entries for which the measured expression level of in the corresponding cell was significantly different. (1st row) For each D. melanogaster query cell, the number of significant expression differences with the nearest target cell in D. yakuba (left) and D. pseudoobscura (right) is shown. (2nd row) The number of significant expression differences score for the best matched cell (smallest expression distance) within the nearest 30 for both D. yakuba and D. pseudoobscura is shown. Numbers above each panel indicate the number of cells that were significantly different at one or more profile entries at a family-wise error rate (FWER) of 0.01. Histograms at bottom show the number cells whose expression profile differed significantly at a given number of entries (genes/time points). Histograms also show a control that compared different D. mel atlases constructed from two disjoint sets of embryos. Expression levels in the paired control atlases are statistically identical at this confidence level for nearly all nuclei. Even after local searching, some cells have statistically different gene expression profiles. As an alternative to the expression distance score (Figure 6), we evaluate the similarity of matched cells using independent pair-wise comparisons for all 11 gene expression levels at each time point. Top panels show the number of expression profile entries for which the measured expression level of in the corresponding cell was significantly different. (1st row) For each D. melanogaster query cell, the number of significant expression differences with the nearest target cell in D. yakuba (left) and D. pseudoobscura (right) is [file pgen.1002346.s009.tif]

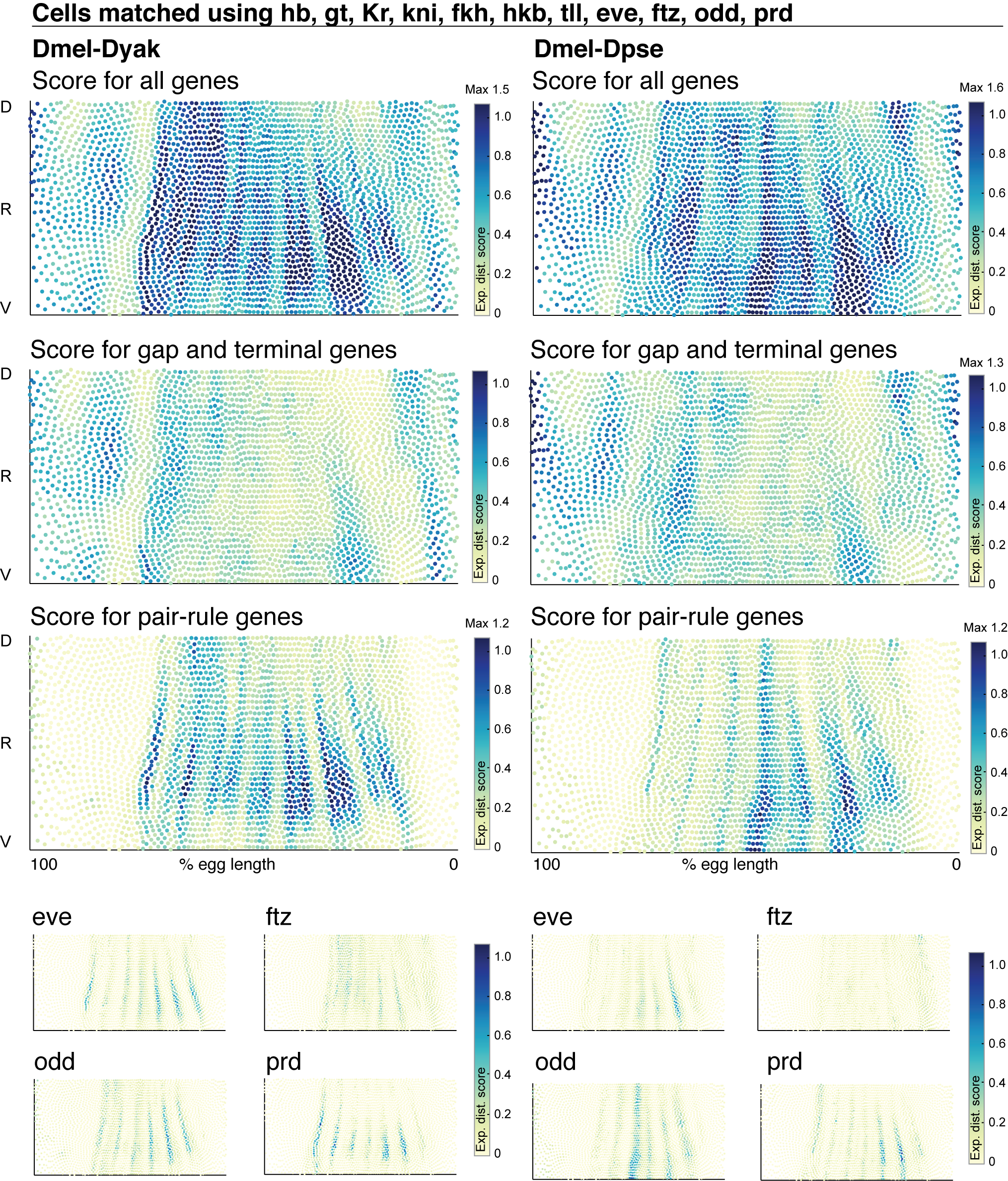

Supplement: Figure S10 — Expression differences are widespread throughout the network. Corresponding cells were identified by searching amongst the nearest 30 cells and scoring the whole gene expression profile (hb, gt, Kr, kni, fkh, hkb, tll, eve, ftz, odd, prd). The contributions to the expression distance score for the best-matched cell were calculated for various tiers of the network (the gap and terminal genes, the pair-rule genes as a group and the individual pair-rule genes). High expression distance scores, indicating poor matches, are darker. All cells scoring above 1.0 are colored the darkest blue; when the maximum value exceeds 1.0, the maximum value amongst all cells is reported at the top of the color map. (TIF) [file pgen.1002346.s010.tif]

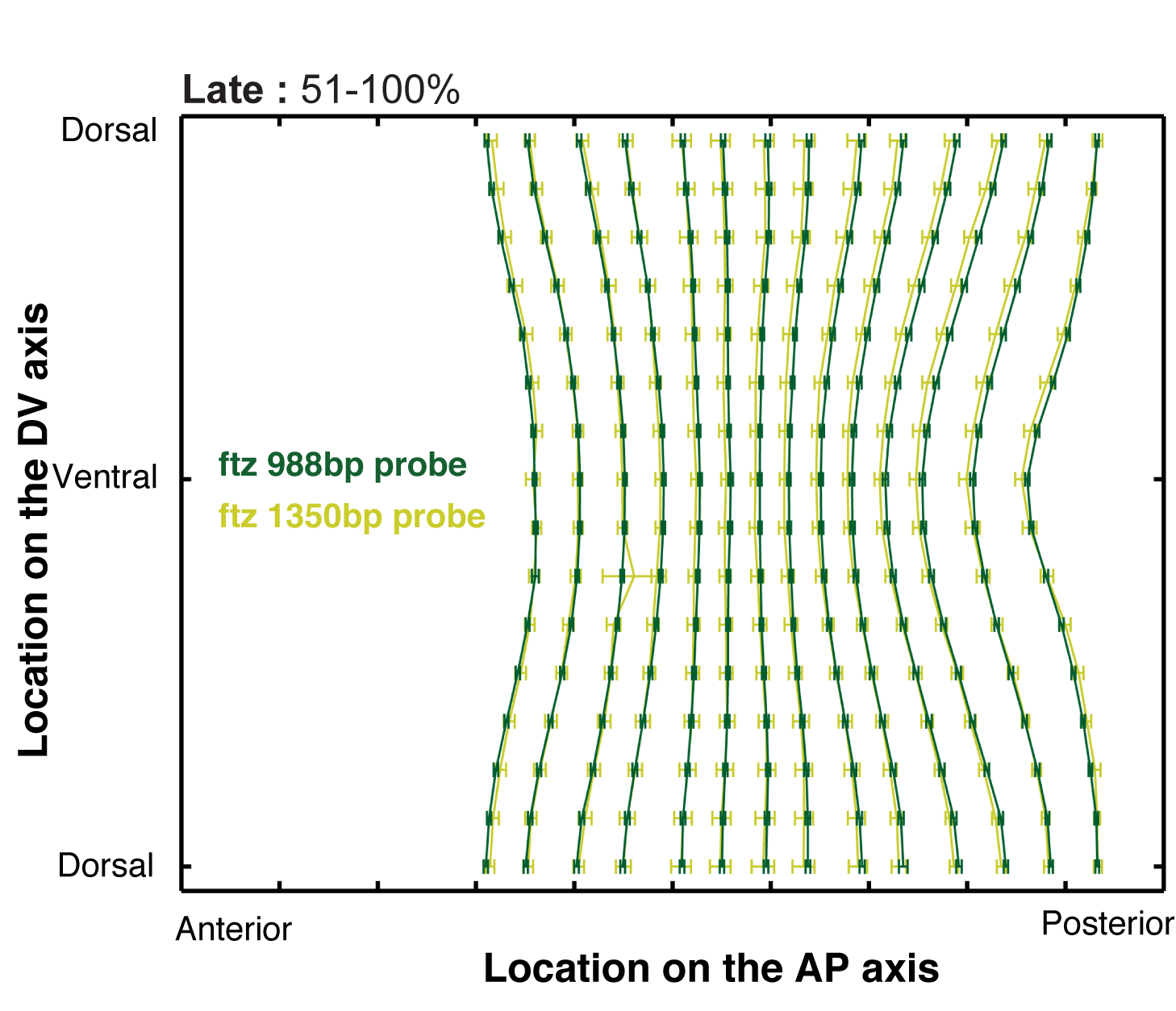

Supplement: Figure S11 — Probe length does not significantly effect position measurements. ftz expression was measured using either a 988 bp exonic (dark green) or a 1350 bp cDNA (light green) in situ probe. Individual pointclouds were divided into 16 dorsal/ventral strips, and the position of the boundaries of ftz expression in each strip was measured. The average position and 95% confidence intervals at each of the 16 positions are plotted for embryos later in cellularization (51–100%). (TIF) [file pgen.1002346.s011.tif]
