# Supplementary material for: A Conserved Developmental Patterning Network Produces Quantitatively Different Output in Multiple Species of Drosophila
Source: PLoS Genet. 2011 Oct 27;7(10):e1002346. doi: 10.1371/journal.pgen.1002346 (PMC3203197; doi:10.1371/journal.pgen.1002346)

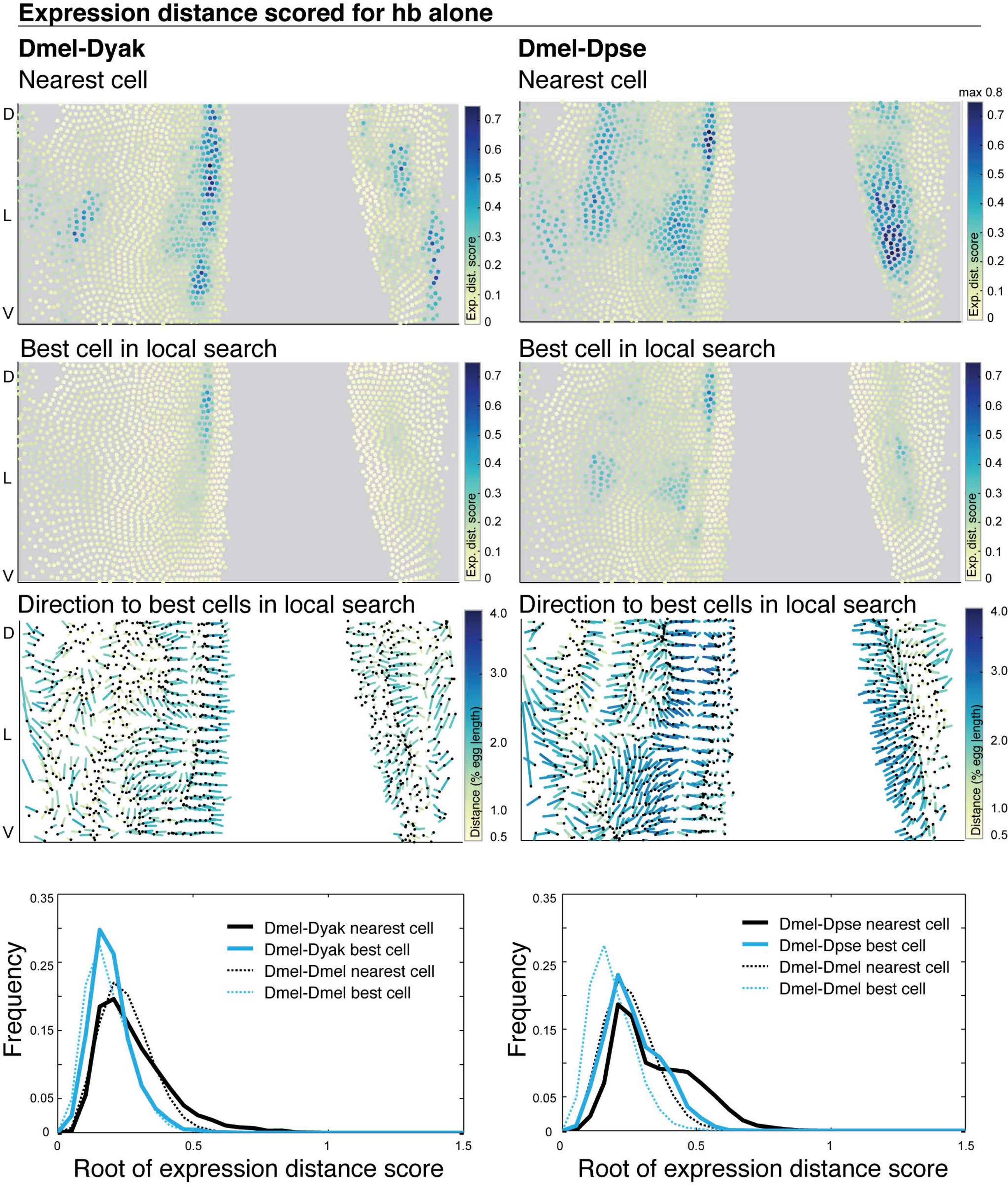

Expression distance scored for hb alone
Dmel-Dyak
Nearest cell
Best cell in local search
Direction to best cells in local search
Dmel-Dpse
Nearest cell
Best cell in local search
Direction to best cells in local search
D
L
V
max 0.8
Exp. dist. score
0
0.1
0.2
0.3
0.4
0.5
0.6
0.7
Distance (% egg length)
0.5
1.0
2.0
3.0
4.0
Frequency
Root of expression distance score
0
0.05
0.15
0.25
0.35
0.5
1
1.5
Dmel-Dyak nearest cell
Dmel-Dyak best cell
Dmel-Dmel nearest cell
Dmel-Dmel best cell
Dmel-Dpse nearest cell
Dmel-Dpse best cell
Dmel-Dmel nearest cell
Dmel-Dmel best cell

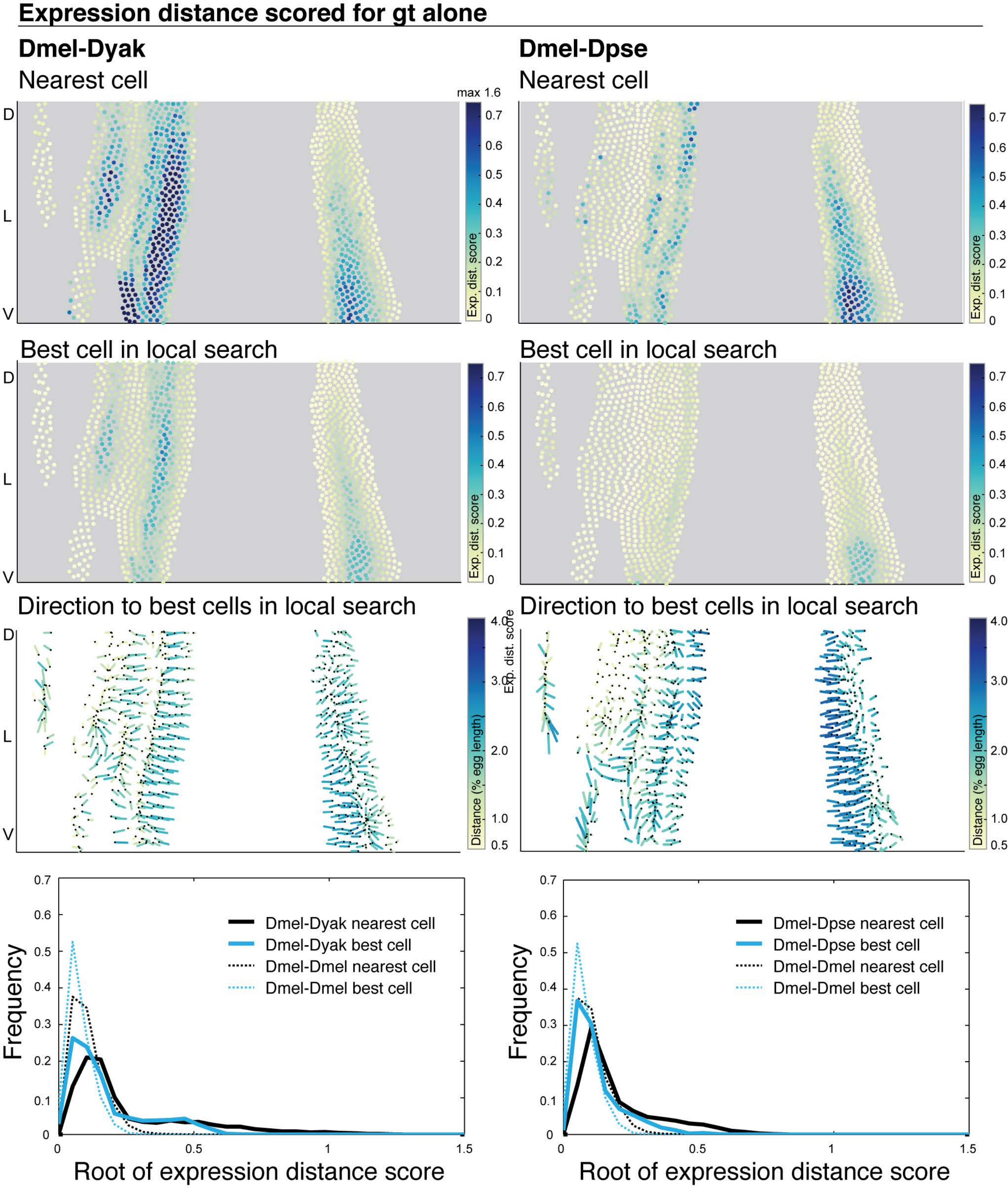

Expression distance scored for gt alone
Dmel-Dyak
Dmel-Dpse
Nearest cell
Best cell in local search
Direction to best cells in local search
D
L
V
max 1.6
Exp. dist. score
Distance (% egg length)
Frequency
Root of expression distance score
Dmel-Dyak nearest cell
Dmel-Dyak best cell
Dmel-Dmel nearest cell
Dmel-Dmel best cell
Dmel-Dpse nearest cell
Dmel-Dpse best cell

Expression distance scored for Kr alone

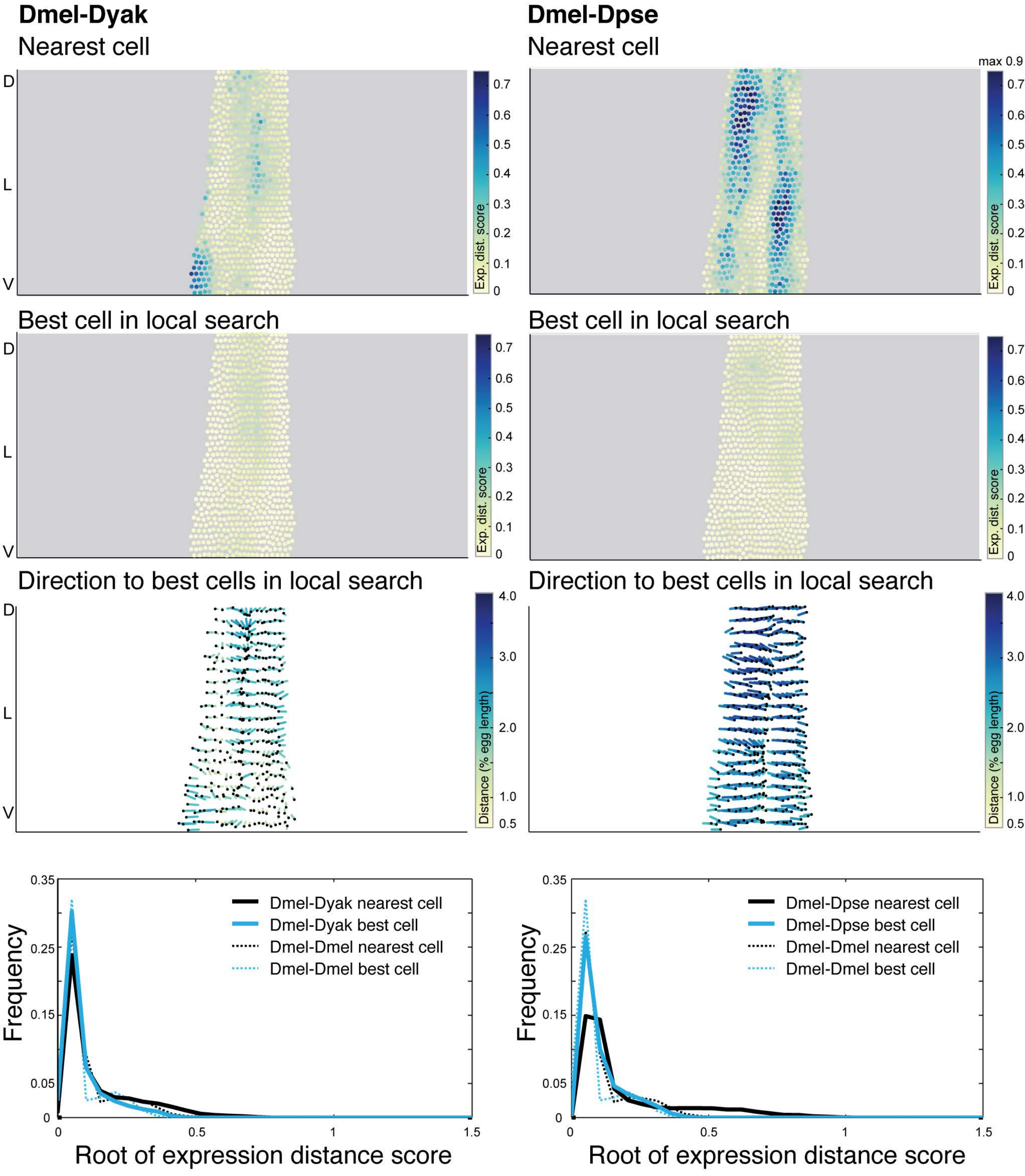

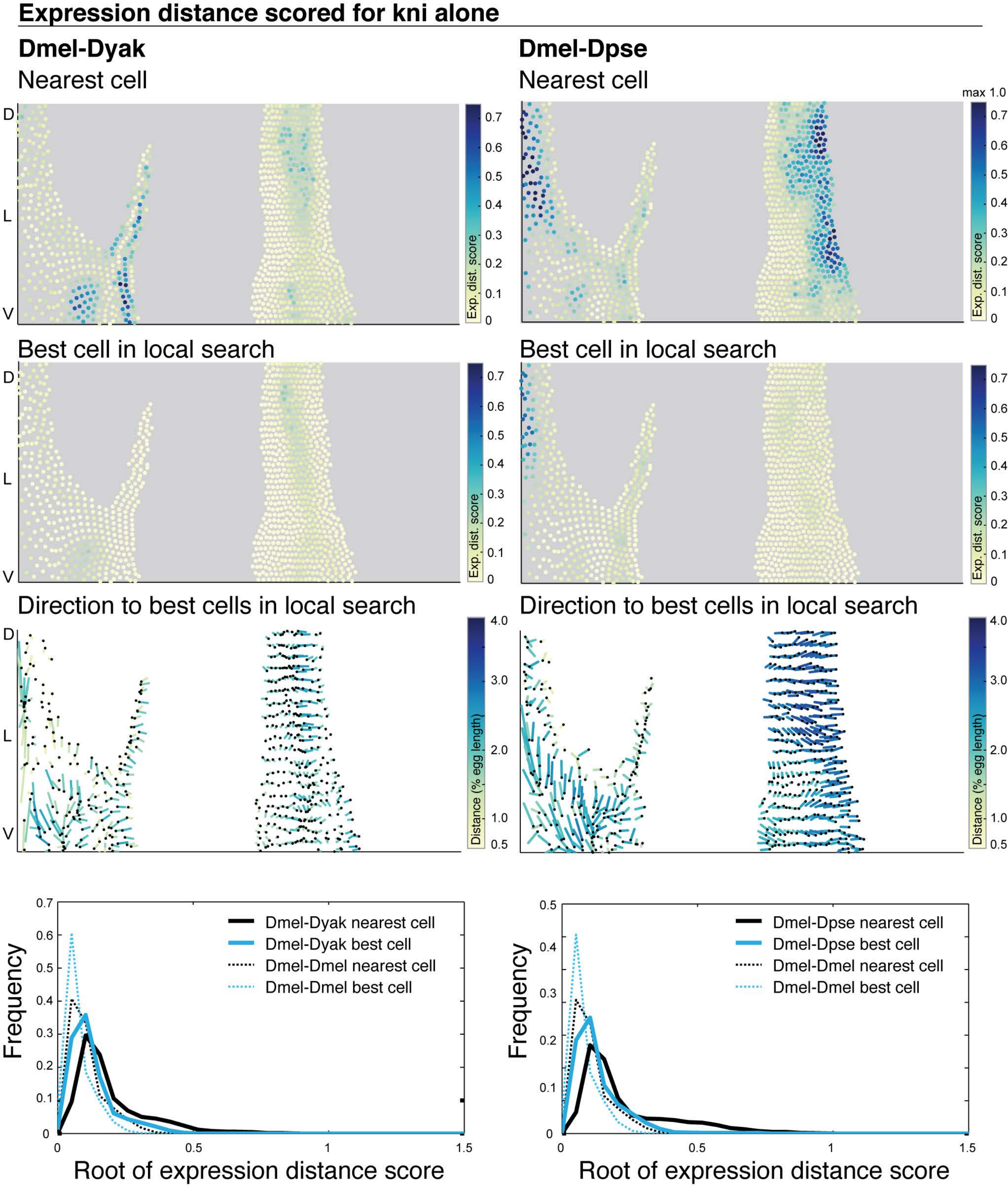
Expression distance scored for kni alone
Dmel-Dyak
Nearest cell
Best cell in local search
Direction to best cells in local search
Dmel-Dpse
Nearest cell
Best cell in local search
Direction to best cells in local search
D
L
V
Exp. dist. score
max 1.0
Distance (% egg length)
Frequency
Root of expression distance score
Dmel-Dyak nearest cell
Dmel-Dyak best cell
Dmel-Dmel nearest cell
Dmel-Dmel best cell
Dmel-Dpse nearest cell
Dmel-Dpse best cell
Dmel-Dmel nearest cell
Dmel-Dmel best cell

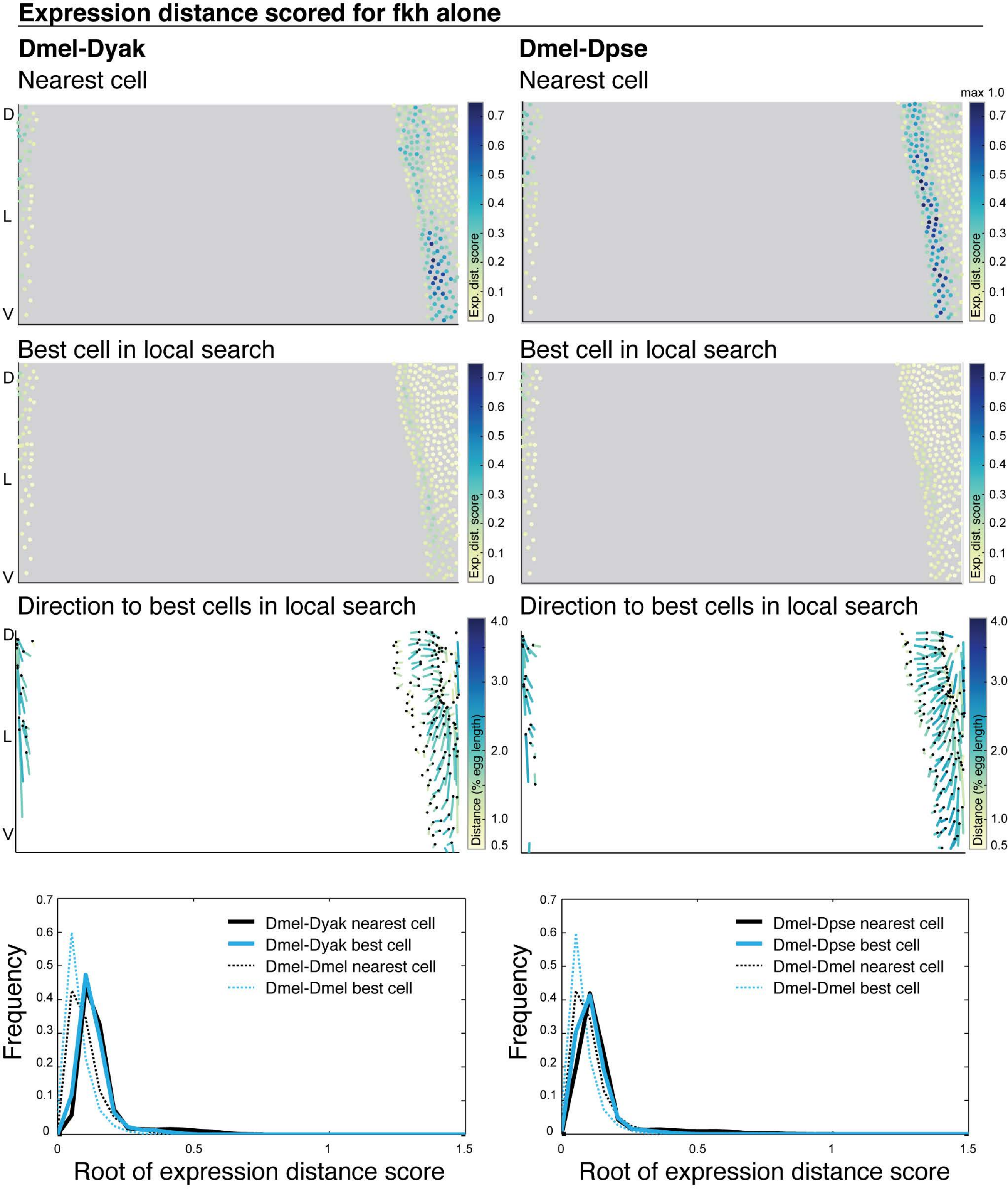

Expression distance scored for fkh alone
Dmel-Dyak
Dmel-Dpse
Nearest cell
Best cell in local search
Direction to best cells in local search
D
L
V
max 1.0
Exp. dist. score
0.7
0.6
0.5
0.4
0.3
0.2
0.1
0
Distance (% egg length)
4.0
3.0
2.0
1.0
0.5
Frequency
Root of expression distance score
Dmel-Dyak nearest cell
Dmel-Dyak best cell
Dmel-Dmel nearest cell
Dmel-Dmel best cell
Dmel-Dpse nearest cell
Dmel-Dpse best cell
0
0.5
1
1.5

Expression distance scored for fkh alone

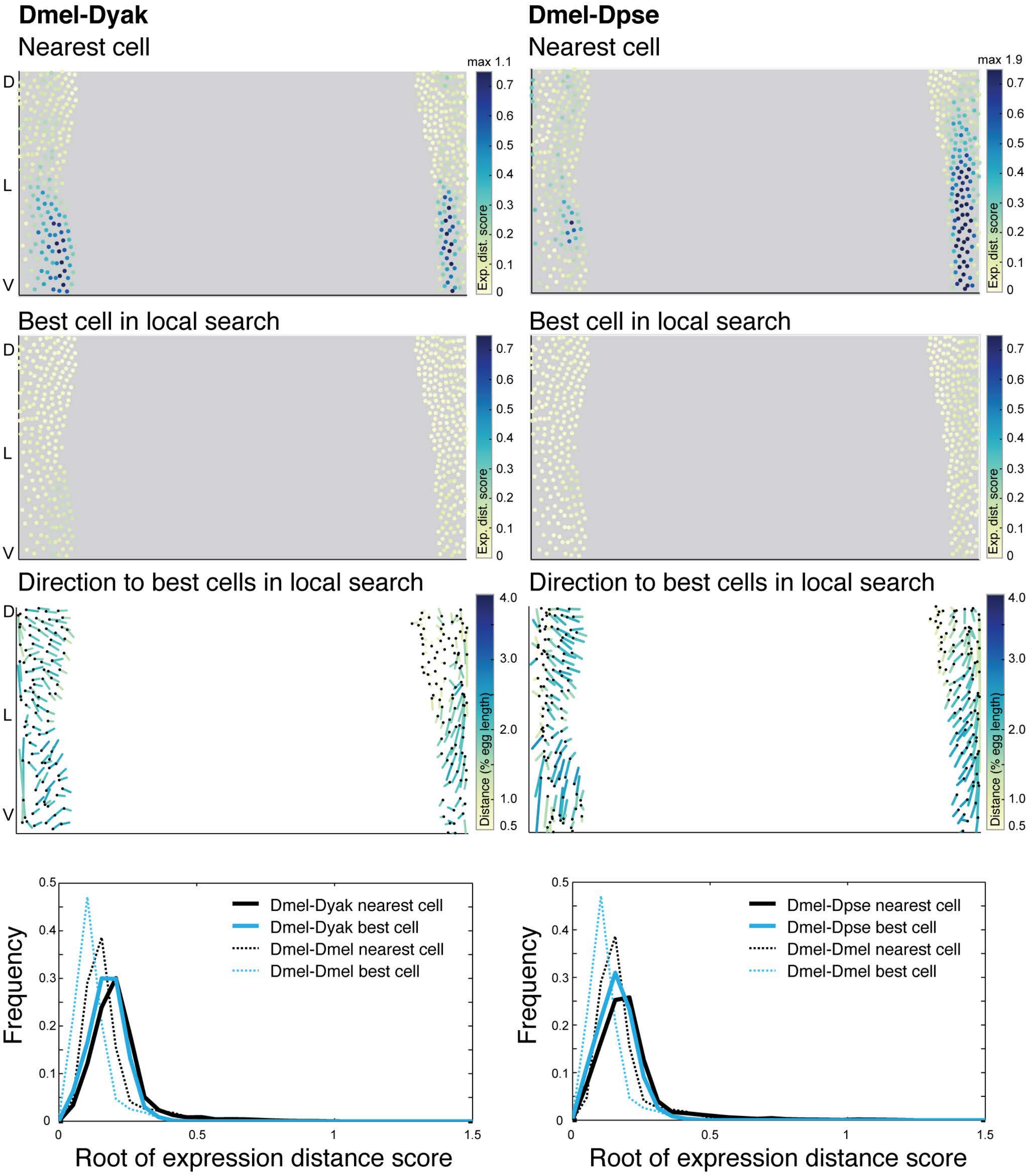

Expression distance scored for tll alone

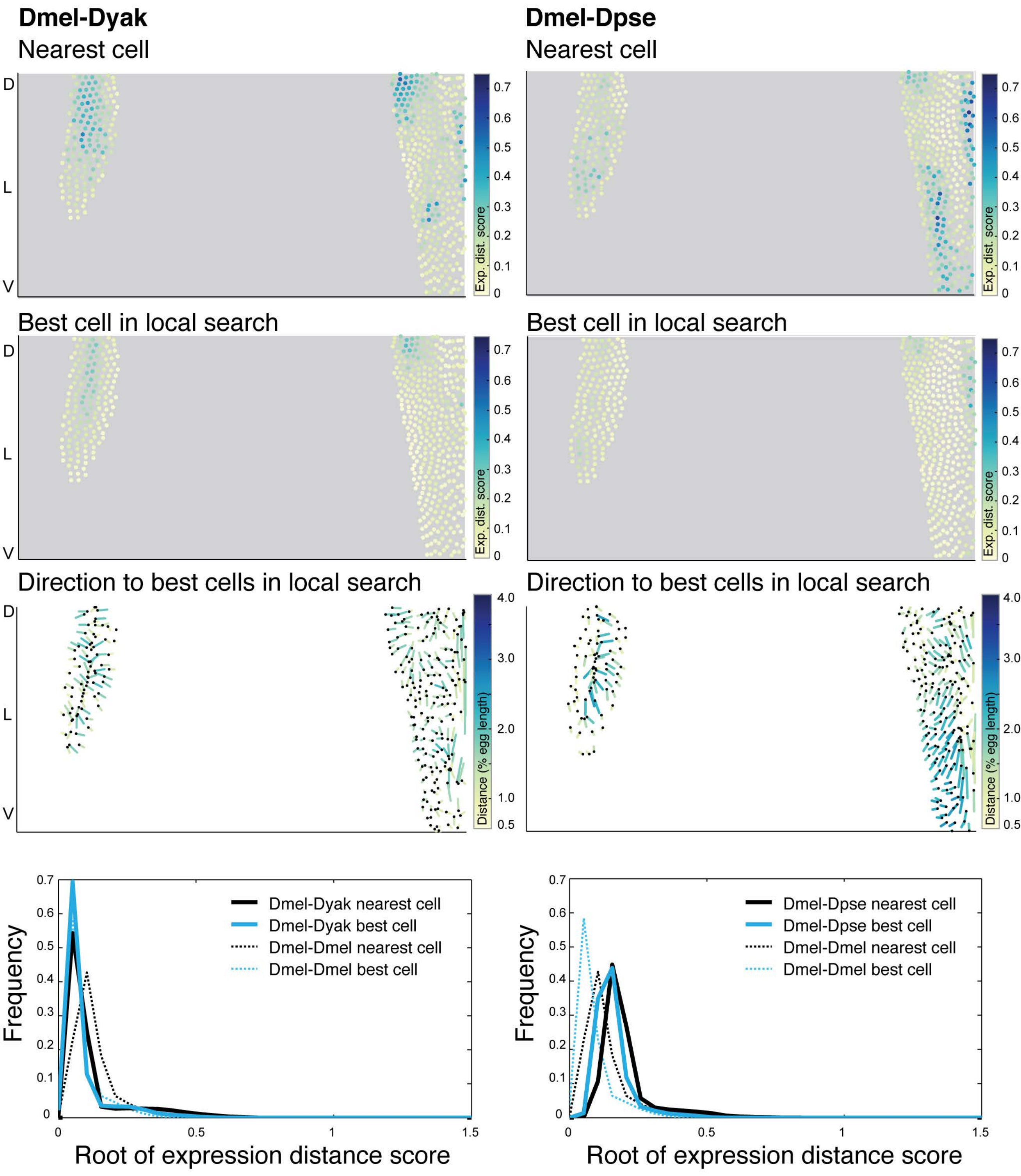

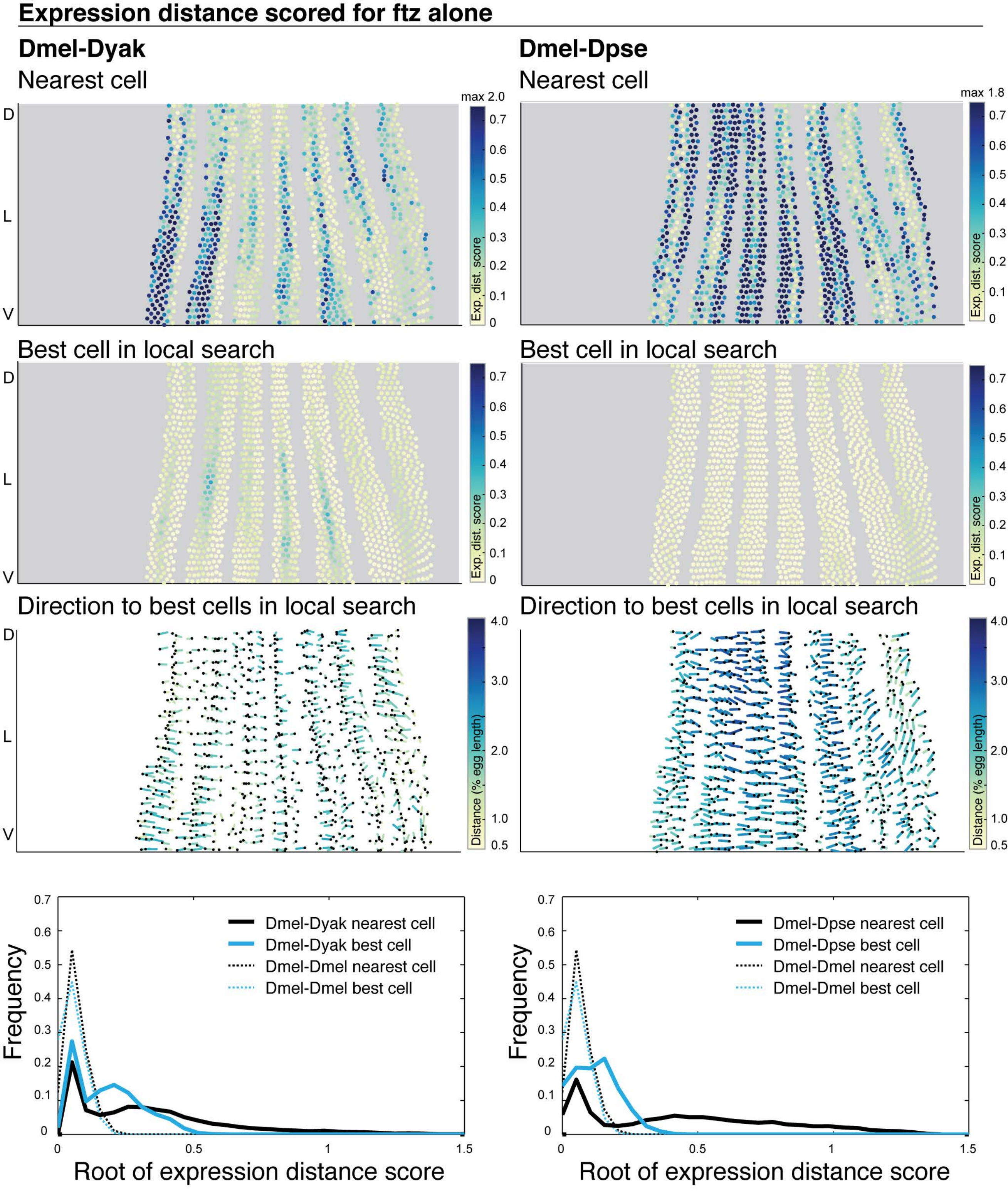

Expression distance scored for ftz alone
Dmel-Dyak
Dmel-Dpse
Nearest cell
max 2.0
max 1.8
D
L
V
Exp. dist. score
0
0.1
0.2
0.3
0.4
0.5
0.6
0.7
Best cell in local search
Direction to best cells in local search
Distance (% egg length)
0.5
1.0
2.0
3.0
4.0
Frequency
Root of expression distance score
Dmel-Dyak nearest cell
Dmel-Dyak best cell
Dmel-Dmel nearest cell
Dmel-Dmel best cell
Dmel-Dpse nearest cell
Dmel-Dpse best cell
0
0.5
1
1.5

Expression distance scored for odd alone

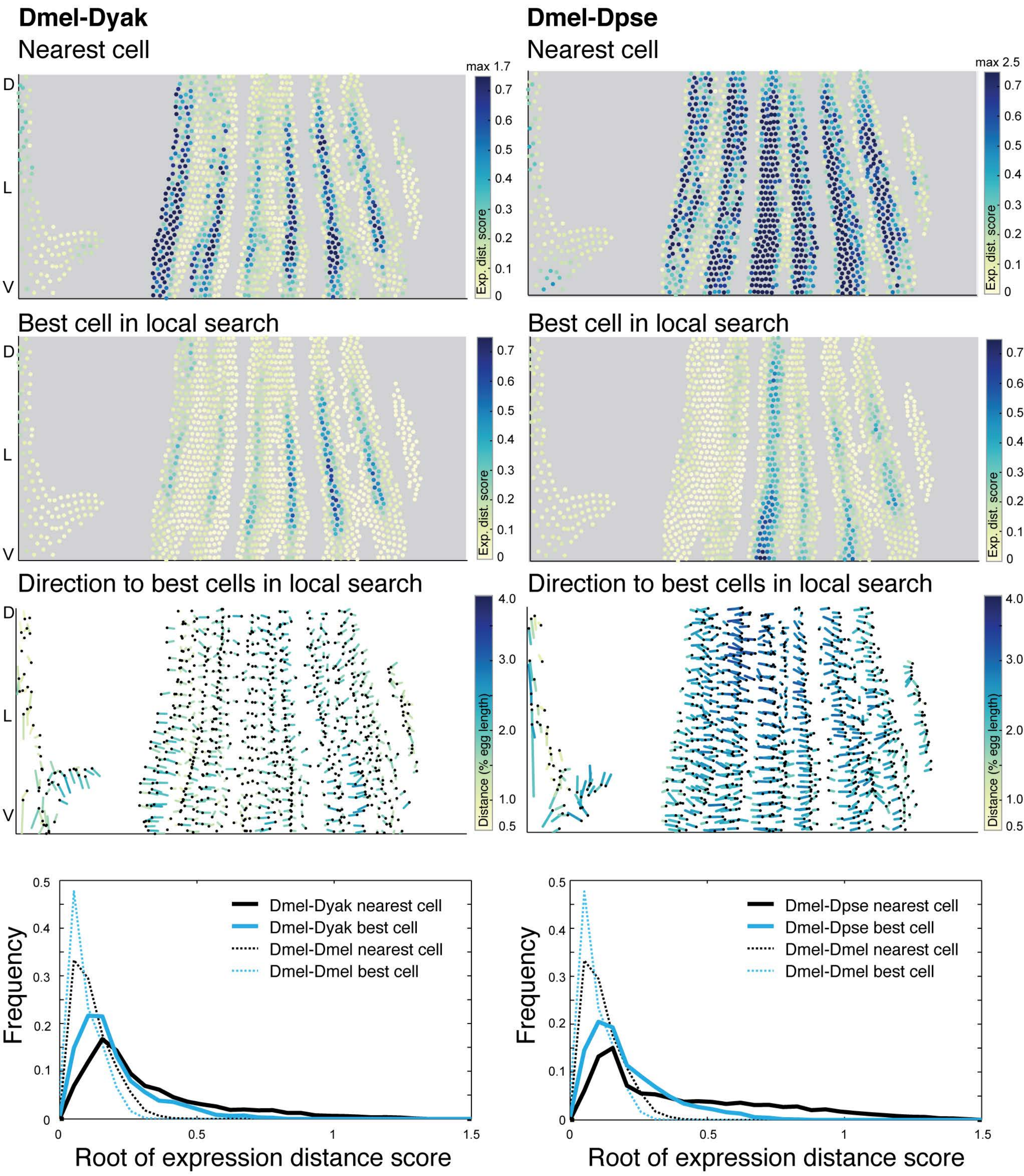

Expression distance scored for prd alone

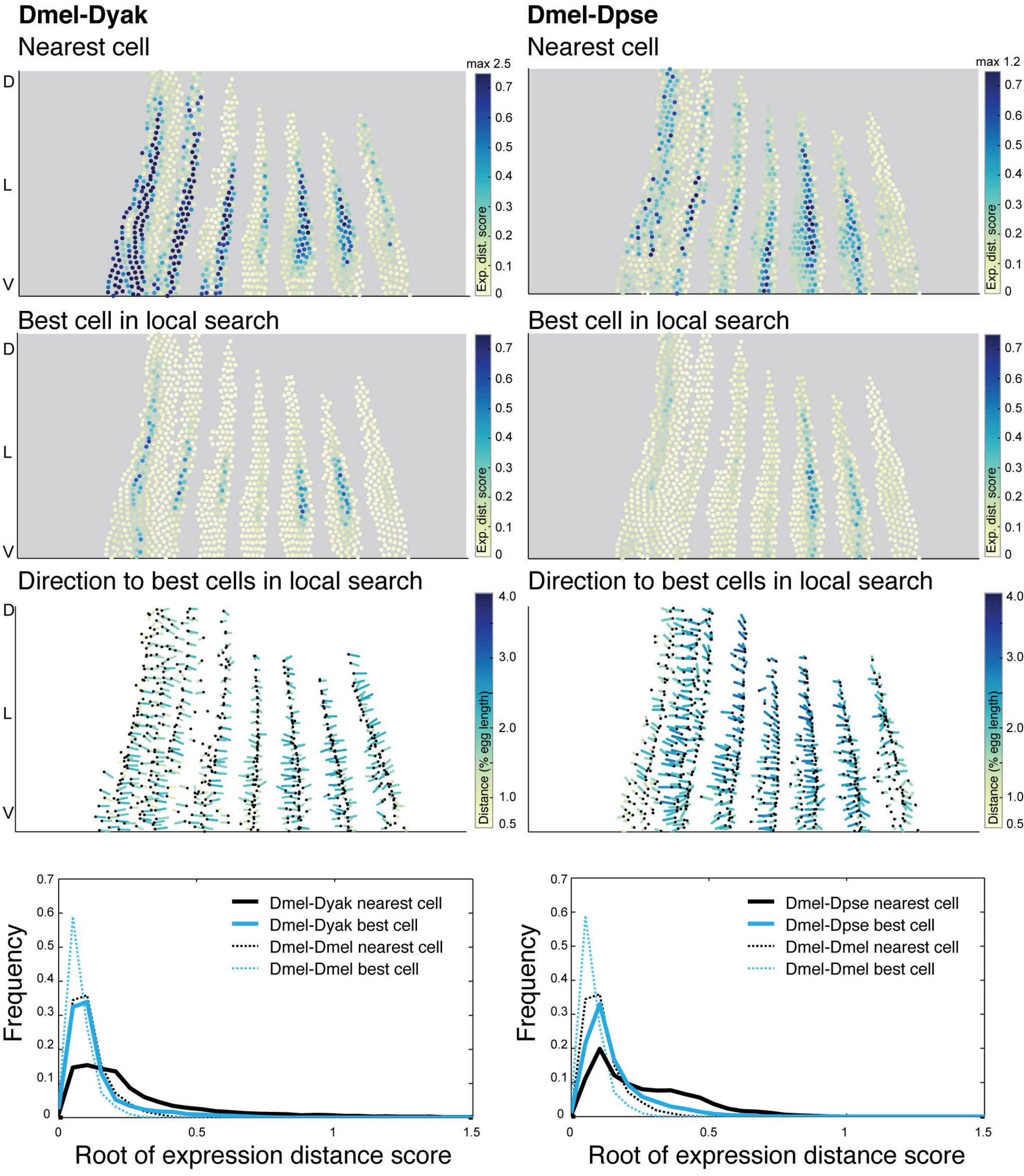

Supplement: Figure S4 — Individual gene expression patterns vary in relative position and intensity. (1st row) For each D. melanogaster query cell, the expression distance score of the nearest target cell in D. yakuba (left) and D. pseudoobscura (right) is shown. (2nd row) The expression distance score for the best-matched cell within the nearest 30 for both D. yakuba and D. pseudoobscura is shown. High expression distance scores, indicating poor matches, are darker. All cells scoring above 0.7 are colored the darkest blue; the maximum value amongst all cells is reported at the top of the color map. (3rd row) For each D. melanogaster query cell, the distance and direction to the average position of the top 10 best corresponding target cells is shown. The correspondence is shown with a line that starts at the position of the query cell, and ends at the average position of the target cells. The end of the line is indicated with a black dot. Because the 2D projection distorts actual distance in 3D, the lines are color-coded to indicate actual distance traversed in 3D. Blue is a large distance, yellow is a small distance. (4th row) The distribution of expression distance scores using only the nearest cell (grey) and best-matched cell within the nearest 30 (blue) are shown. The distribution of scores narrows and the mode decreases after a local search. To establish the significance of the calculated differences, we assembled two atlases from the D. melanogaster dataset, and compared these two atlases to each other (dotted lines). We show data from fkh, ftz, gt, hb, hkb, kni, Kr, odd, prd and tll, which together with eve (described in Figure 5) form the set of 11 genes used for analyzing the whole gene expression profile. (PDF) [file pgen.1002346.s004.pdf]
