# Supplementary material for: A Conserved Developmental Patterning Network Produces Quantitatively Different Output in Multiple Species of Drosophila
Source: PLoS Genet. 2011 Oct 27;7(10):e1002346. doi: 10.1371/journal.pgen.1002346 (PMC3203197; doi:10.1371/journal.pgen.1002346)
Supplement: Table S1 — Number of embryos per cohort in D. yakuba and D. pseudoobscura datasets. The number of embryos per gene varies across the dataset with a minimum of 4 embryos per gene per time point. Data was not collected for time points when the gene is not expressed (i.e. Bcd at later time points). We collected additional data on the registration genes eve and ftz in both species. (DOC) [file pgen.1002346.s012.doc]

**Table S1 : Number of embryos per cohort in *D.yak* and *D.pse*** datasets

| **Gene** | **Species** | **5:0-3%** | **5:4-8%** | **5:9-25%** | **5:26-50%** | **5:51-75%** | **5:76-100%** |
| --- | --- | --- | --- | --- | --- | --- | --- |
| bcd | *D.yak* | 6 | 7 | 3 | 0 | 0 | 0 |
| cad | *D.yak* | 5 | 5 | 9 | 4 | 5 | 7 |
| kni | *D.yak* | 6 | 7 | 6 | 19 | 11 | 8 |
| Kr | *D.yak* | 6 | 4 | 6 | 8 | 5 | 11 |
| gt | *D.yak* | 7 | 6 | 5 | 5 | 6 | 5 |
| hb | *D.yak* | 8 | 9 | 6 | 6 | 5 | 8 |
| tll | *D.yak* | 5 | 7 | 13 | 11 | 7 | 9 |
| hkb | *D.yak* | 5 | 5 | 6 | 8 | 7 | 7 |
| fkh | *D.yak* | 6 | 6 | 7 | 6 | 4 | 13 |
| eve | *D.yak* | 17 | 13 | 21 | 7 | 4 | 5 |
| ftz | *D.yak* | 28 | 20 | 21 | 15 | 9 | 10 |
| odd | *D.yak* | 8 | 10 | 12 | 6 | 8 | 10 |
| prd | *D.yak* | 6 | 6 | 5 | 5 | 5 | 9 |
| bcd | *D.pse* | 7 | 7 | 9 | 0 | 0 | 0 |
| kni | *D.pse* | 7 | 6 | 4 | 4 | 5 | 10 |
| Kr | *D.pse* | 6 | 7 | 7 | 3 | 7 | 7 |
| gt | *D.pse* | 5 | 5 | 7 | 7 | 6 | 4 |
| hb | *D.pse* | 14 | 12 | 11 | 5 | 6 | 12 |
| tll | *D.pse* | 7 | 4 | 4 | 5 | 6 | 4 |
| hkb | *D.pse* | 6 | 6 | 5 | 4 | 6 | 7 |
| fkh | *D.pse* | 3 | 5 | 3 | 5 | 5 | 17 |
| eve | *D.pse* | 43 | 92 | 111 | 78 | 27 | 32 |
| ftz | *D.pse* | 17 | 23 | 34 | 33 | 20 | 33 |
| odd | *D.pse* | 11 | 6 | 11 | 6 | 8 | 10 |
| prd | *D.pse* | 6 | 5 | 14 | 9 | 5 | 7 |
