# Supplementary material for: A Conserved Developmental Patterning Network Produces Quantitatively Different Output in Multiple Species of Drosophila
Source: PLoS Genet. 2011 Oct 27;7(10):e1002346. doi: 10.1371/journal.pgen.1002346 (PMC3203197; doi:10.1371/journal.pgen.1002346)
Supplement: Table S2 — Standard deviation of gene expression is reduced after registration to similar levels in D. melanogaster, D. yakuba, and D. pseudoobscura atlases. The average standard deviation after registration is shown for all genes in the D. yakuba and D. pseudoobscura atlases, as well as the corresponding data from the D. melanogaster atlas for comparison [7]. (DOC) [file pgen.1002346.s013.doc]

| **Average Standard Deviation across cohorts** | | | |
| --- | --- | --- | --- |
| **Gene** | ***D.mel*** | ***D.yak*** | ***D.pse*** |
| bcd | 0.071 | 0.02 | 0.026 |
| cad | 0.165 | 0.094 | x |
| eve | 0.129 | 0.109 | 0.098 |
| fkh | 0.068 | 0.032 | 0.031 |
| ftz | 0.131 | 0.178 | 0.119 |
| gt | 0.108 | 0.067 | 0.087 |
| hb | 0.134 | 0.096 | 0.076 |
| hkb | 0.106 | 0.049 | 0.051 |
| kni | 0.099 | 0.09 | 0.105 |
| Kr | 0.066 | 0.061 | 0.072 |
| odd | 0.119 | 0.125 | 0.08 |
| prd | 0.088 | 0.077 | 0.057 |
| tll | 0.063 | 0.044 | 0.067 |
