# Supplementary material for: A Conserved Developmental Patterning Network Produces Quantitatively Different Output in Multiple Species of Drosophila
Source: PLoS Genet. 2011 Oct 27;7(10):e1002346. doi: 10.1371/journal.pgen.1002346 (PMC3203197; doi:10.1371/journal.pgen.1002346)
Supplement: Table S3 — Mean and median expression distance score before and after local search. A local search significantly decreases the expression distance score relative to direct spatial mapping. The mean and median of the expression distance score using direct spatial mapping (nearest cell) and local search (local search) is shown for pair-wise comparisons between D. melanogaster and D. yakuba and D. melanogaster and D. pseudoobscura, for both individual genes and all genes in our dataset. (DOC) [file pgen.1002346.s014.doc]

| species & gene(s) | mean :nearest cell | mean :local seach | median :nearest cell | median :local search |
| --- | --- | --- | --- | --- |
| D.yak / hb | 0.09307 | 0.04595 | 0.06264 | 0.03462 |
| D.yak / gt | 0.11102 | 0.04903 | 0.02852 | 0.01454 |
| D.yak / Kr | 0.03686 | 0.01454 | 0.00621 | 0.00355 |
| D.yak / kni | 0.05241 | 0.02199 | 0.02242 | 0.01087 |
| D.yak / fkh | 0.03416 | 0.02070 | 0.01687 | 0.01440 |
| D.yak / hkb | 0.05651 | 0.03481 | 0.03988 | 0.03124 |
| D.yak / tll | 0.02867 | 0.01511 | 0.00540 | 0.00357 |
| D.yak / eve | 0.15759 | 0.04785 | 0.05745 | 0.01429 |
| D.yak / ftz | 0.17556 | 0.04817 | 0.08724 | 0.03017 |
| D.yak / odd | 0.14615 | 0.05687 | 0.05668 | 0.02484 |
| D.yak / prd | 0.12924 | 0.03103 | 0.03752 | 0.00951 |
| D.yak/all genes | 1.02124 | 0.57477 | 0.78574 | 0.53324 |
| D.pse / hb | 0.14056 | 0.08066 | 0.09624 | 0.06016 |
| D.pse / gt | 0.06312 | 0.02349 | 0.02096 | 0.00868 |
| D.pse / Kr | 0.07410 | 0.01757 | 0.01085 | 0.00539 |
| D.pse / kni | 0.08259 | 0.02592 | 0.02699 | 0.01123 |
| D.pse / fkh | 0.03704 | 0.01528 | 0.01304 | 0.00958 |
| D.pse / hkb | 0.06067 | 0.03002 | 0.03307 | 0.02510 |
| D.pse / tll | 0.05035 | 0.02790 | 0.03081 | 0.02030 |
| D.pse / eve | 0.20313 | 0.03619 | 0.08667 | 0.01344 |
| D.pse / ftz | 0.31421 | 0.02208 | 0.17765 | 0.01445 |
| D.pse / odd | 0.27256 | 0.06548 | 0.09802 | 0.02704 |
| D.pse / prd | 0.09772 | 0.03619 | 0.04536 | 0.01433 |
| D.pse / all genes | 1.39606 | 0.58152 | 1.13876 | 0.55014 |
