# Supplementary material for: A Conserved Developmental Patterning Network Produces Quantitatively Different Output in Multiple Species of Drosophila
Source: PLoS Genet. 2011 Oct 27;7(10):e1002346. doi: 10.1371/journal.pgen.1002346 (PMC3203197; doi:10.1371/journal.pgen.1002346)
Supplement: Table S4 — Probe constructs used for D. yakuba and D. pseudoobscura in situ hybridizations. The gene, species, template type (cDNA or genomic DNA (gDNA), length, and primers are shown for each in situ probe used in this study. (DOC) [file pgen.1002346.s015.doc]

| **Gene** | **Species** | **Source** | **Length (bp)** | **5' (Left) Primer** | **3' (Right) Primer** |
| --- | --- | --- | --- | --- | --- |
| bcd | *d.yak* | cDNA | 2771 | TACCATCATCCGCTGCCCCACACACA | GCCCGCGGCGTTACGATGGGGATTAT |
| cad | *d.yak* | cDNA | 1288 | GCCCTACACACAAAAGCACAGTGCCGC | TCACATCGAGAGCGTGCCCACTGCC |
| eve | *d.yak* | gDNA | 531 | TACCGCACCTACAACATGGA | TCTTGGCGTCCGATAAAATC |
| fkh | *d.yak* | cDNA | 1321 | TCCGCCTACACAATGAACAG | GCGCATGATAGCCAAGACTC |
| ftz | *d.yak* | cDNA | 1362 | CCAGAGCCACTACAGCTACGCCGACA | TGGTAGCTGCACTGCTGTTGGTACTGC |
| Gt | *d.yak* | gDNA | 691 | CAGCAACGCCAGCACCAT | TTAGGCGGTGGTTACTTTGG |
| hb | *d.yak* | cDNA | 2178 | ACAGCCACGACCAACTACGAGCAGCA | ATCGTCGCAGCTGTGGTAGCCCATGT |
| hkb | *d.yak* | cDNA | 918 | AACCTGCATCCACCGCAAACCTACGC | GTACATGGGCACGAAGATGGACGGGC |
| Kni | *d.yak* | gDNA | 1069 | GCAAGGCGTGCCGCTTGAGGAAGTGCT | GCCACCGCGAAGCCACCGAAGTGAGTG |
| Kr | *d.yak* | cDNA | 1482 | TGCTTCAAGACGCACAAACGCGAACC | GGCCCATATAAGGAGCAGCGGCGTCA |
| odd | *d.yak* | gDNA | 1281 | CAGCAACATAACCGTGGATG | GCTCGAGGATGACGAGGATA |
| prd | *d.yak* | gDNA | 1673 | GCAATGCACAGACCCTTCTT | CGTGTCATAGCTGGAGACGA |
| tll | *d.yak* | cDNA | 1403 | AATACAACAGCGTGCGCCTTTCGCCA | ATGGTGATGTCGCCGATGGTCTTGCG |
| bcd | *d.pse* | gDNA | 1741 | GGCAAACTGTGTGTTGCCTCCGGCA | TCCCGCATCCTCATCCCCACCATCCTC |
| cad | *d.pse* | gDNA | 750 | TGGTTTCACACTACTACAATACACTGC | GATGAAGAAGCCCGCATATC |
| eve | *d.pse* | cDNA | 1147 | ATGCACGGATACCGAACATACAACATGG | CGCCTCAGTCTTGTAGGGTTTGAAGAG |
| fkh | *d.pse* | cDNA | 1400 | GTGGCATGTCCTCGGTATCT | TGCTGTGGTTGGTGTGGTAT |
| ftz | *d.pse* | cDNA | 1350 | CACAGCCTGCCGCCCACGTACTATGA | TGACACTGCACTGCTGCTGGGTCTGT |
| Gt | *d.pse* | gDNA | 1518 | GCTGATGCACGAGAAACTCA | CAGATTGATGCGCTCAAG |
| hb | *d.pse* | cDNA | 1871 | GGAATCACTTGGAGCAGTACCT | ACAATGTTCTTCGGGTAGCTGT |
| hkb | *d.pse* | cDNA | 889 | TGTCGACTATTAACCTGCATCC | GGCATATGCGTCTTCATGTG |
| Kni | *d.pse* | gDNA | 1120 | CATCTGCACCACCAGCAC | ACACAAAGATACCCCGCATC |
| Kr | *d.pse* | cDNA | 1133 | ATGGCTATATCAATGCTCCAGGACGCACA | CGCCTCAGTCTTGTAGGGTTTGAAGAG |
| odd | *d.pse* | gDNA | 1360 | CGCCGATCAGCAACATAAC | ATTTCGTCGATGGTGAAACC |
| prd | *d.pse* | cDNA | 1745 | AATGCACAGACCCTTCTTCAAT | GACTCGTTGGGACTCTCGTTAC |
| tll | *d.pse* | cDNA | 1178 | GGGAAGCACTACGGCATCTA | GTCTTGCGGAAGAACAGCTC |
